# Stepwise PEG synthesis featuring deprotection and coupling in one pot

**DOI:** 10.3762/bjoc.17.207

**Published:** 2021-12-28

**Authors:** Logan Mikesell, Dhananjani N A M Eriyagama, Yipeng Yin, Bao-Yuan Lu, Shiyue Fang

**Affiliations:** 1 Department of Chemistry, Michigan Technological University, 1400 Townsend Drive, Houghton, MI 49931, USA; 2 ChampionX, 11177 South Stadium Drive, Sugar Land, TX 77478, USA

**Keywords:** base-labile, monodisperse, PEG, polyethylene glycol, protecting group

## Abstract

The stepwise synthesis of monodisperse polyethylene glycols (PEGs) and their derivatives usually involves using an acid-labile protecting group such as DMTr and coupling the two PEG moieties together under basic Williamson ether formation conditions. Using this approach, each elongation of PEG is achieved in three steps – deprotection, deprotonation and coupling – in two pots. Here, we report a more convenient approach for PEG synthesis featuring the use of a base-labile protecting group such as the phenethyl group. Using this approach, each elongation of PEG can be achieved in two steps – deprotection and coupling – in only one pot. The deprotonation step, and the isolation and purification of the intermediate product after deprotection using existing approaches are no longer needed when the one-pot approach is used. Because the stepwise PEG synthesis usually requires multiple PEG elongation cycles, the new PEG synthesis method is expected to significantly lower PEG synthesis cost.

## Introduction

Polyethylene glycols and derivatives (PEGs) have found wide applications in many areas [1–6]. For some applications, polydisperse PEGs are acceptable although those with narrow molecular weight distribution are always desirable. These PEGs can be synthesized conveniently by polymerization of ethylene oxide under basic or acidic conditions [7]. The polymerization methods are inexpensive and PEGs with high molecular weight can be obtained. However, for many other applications, which include as linkers in organic synthesis and bioconjugation [8], as ingredients in nanomedicines to stabilize nanoparticles and to assist nanoparticle cell entry [9–11], and as PEGylation agents to stabilize drugs based on biologic molecules such as peptides, proteins and nucleic acids and to evade undesired immune responses, monodisperse PEGs are required or highly desired [12–13].

**Scheme 1:**
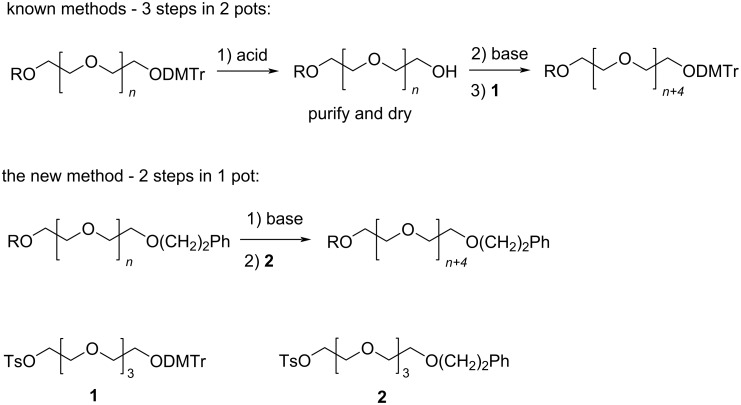
A comparison of the new PEG synthesis method with a typical known PEG synthesis method.

To meet the needs of monodisperse PEGs, significant efforts have been made to develop stepwise methods for their synthesis [14–26]. Perhaps, the most widely used methods in academia and in industry involve the use of monomers such as compound **1**, which contain the acid-labile DMTr protecting group. PEG elongation is achieved by deprotection under acidic conditions, purifying the intermediate, and setting up a separate reaction to carry out the deprotonation and Williamson ether formation reactions under basic conditions (Scheme 1) [15–16,18,23,25]. It is remarkable that the method has evolved to such a sophistication that the synthesis of (PEG)_16_ was achieved in nine steps without any chromatography [18,27]. Besides PEGs, similar approaches have also been used for the synthesis of oligosulfoxides [28]. In this article, we report the use of monomers such as **2** containing a base-labile protecting group with the phenethyl group for stepwise monodisperse PEG synthesis. With monomers having a base-labile protection group, PEG elongation is achieved in two steps – deprotection and coupling – in only one pot (Scheme 1). There is no need to isolate and purify the intermediate between deprotection and coupling, and the deprotonation step is not needed. Our results show that the synthesis is far more convenient than known methods, and high quality of monodisperse PEGs can be obtained in acceptable to high yields.

## Results and Discussion

**Scheme 2:**
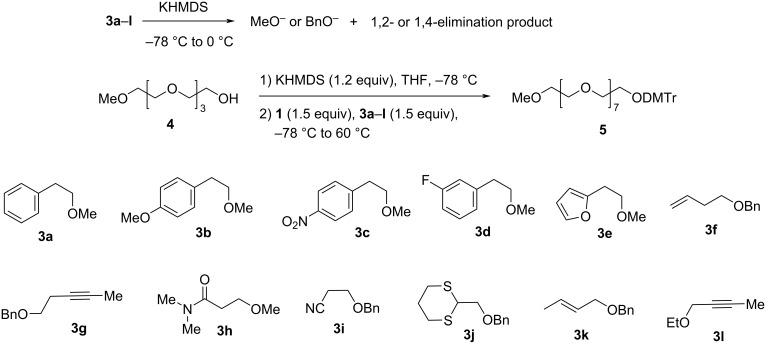
Screening base-labile protecting groups for stepwise PEG synthesis. All compounds (**3a**–**l**) underwent 1,2- or 1,4-elimination in the presence of KHMDS at 0 °C. Except for compound **3h**, all other compounds were found stable under the basic Williamson ether formation reaction conditions between compounds **4** and **1**.

For a base-labile protecting group to be useful in PEG synthesis using the one-pot PEG elongation approach, it needs to meet two criteria: (1) The protecting group can be removed under basic conditions. (2) The protecting group is stable under the basic Williamson ether formation conditions. For this reason, we screened several potentially useful protecting groups against these two criteria using compounds **3a**–**l** (Scheme 2). For criterion (1), we subjected the compounds into basic conditions and used TLC to monitor the progress of the 1,2-elimination (**3a**–**j**) or 1,4-elimination (**3k**–**l**) reactions. Initially, compound **3a** (1 equiv) was treated with LDA (1 equiv) with catalytic amount of *t-*BuOK (0.1 equiv) in THF at −78 °C [29–30]. Complete consumption of **3a** to give methoxide and styrene was observed after warming the reaction mixture to −50 °C and stirring at the temperature for less than two hours. Because LDA has a short shelf life, and has to be stored at low temperature, we were curious if KHMDS (p*K*_a_ of conjugate acid, 26), which is a much weaker base than LDA (p*K*_a_ of conjugate acid, 36) [31] and can be stored at room temperature for a long period of time, could also bring about the reaction. Surprisingly, we found that the reaction occurred with high efficiency even without using any catalysts. Therefore, KHMDS was used for screening the rest of the compounds (**3b**–**l**). Gratifyingly, all the compounds underwent 1,2-elimination (**3b**–**j**) or 1,4-elimination (**3k**–**l**) readily using this weaker base, and according to TLC (Supporting Information File 1), the reactions had 100% conversion after stirring at 0 °C for less than two hours (Scheme 2). Thus, we concluded that all the protecting groups in compounds **3a**–**l** meet the criterion (1).

For criterion (2), we conducted the Williamson ether formation reaction between compounds **4** and **1** to form compound **5** using KHMDS as the base in the presence of compounds **3a**–**l**. Compound **4** (1 equiv) in THF was deprotonated with KHMDS (1.2 equiv). The mixture was cooled to −78 °C, and the solution of **1** (1.5 equiv) and **3a**–**k** or **3l** (1.5 equiv) in THF was added. The reaction mixture was warmed to room temperature gradually, and then heated to 60 °C. TLC analysis was performed to determine if the product **5** could be formed without causing the 1,2- or 1,4-elimination reactions of **3**. The addition of excess base for the deprotonation of **4** was to ensure complete deprotonation in the event of inadvertent moisture. Cooling the solution of the deprotonated **4** to low temperature before addition of **1** and **3** and gradually warming the mixture to room temperature before heating was to prevent the removal of the base-labile protecting group in **3** by the excess strong base by allowing the excess base to be consumed selectively via β-elimination of the tosylate in **1**. The product of premature removal of the base-labile protecting group – an alkoxide – would complicate the reaction, while the product of β-elimination of the tosylate – a vinyl ether – is inert under the reaction conditions. Compounds **3a**–**l** were subjected to the study. All the compounds except **3h** were found to be stable under the coupling conditions while product **5** was formed as indicated by TLC analysis (Supporting Information File 1), and the protection groups in them meet criterion (2). In the case of **3h**, the compound was consumed under the Williamson ether formation conditions indicating that the 3-(dimethylamino)-3-oxopropyl group in it does not meet criterion (2). Based on the results of screening compounds **3a**–**l** against criteria (1) and (2), we concluded that the protecting groups in compounds **3a**–**g** and **3i**–**l** can be used as the base-labile protection group for the new PEG synthesis method featuring PEG elongation in one-pot.

Among the groups studied, the phenethyl group (i.e., -(CH_2_)_2_Ph) is one of the simplest. In addition, when the proposed one-pot PEG elongation approach is used for the synthesis of long PEGs, higher temperature and longer reaction time are usually needed for the Williamson ether formation reaction [23]. This requires the protecting groups to be stable under conditions harsher than those used in our screening studies. Therefore, it is preferable to choose a relatively more stable group than a less stable one for the one-pot PEG elongation application. Among the groups studied, the phenethyl group was conjectured to belong to the more stable ones. With these considerations, the phenethyl group was chosen for the development of the one-pot PEG elongation approach for PEG synthesis although other groups that meet the two criteria can be used as well.

**Scheme 3:**
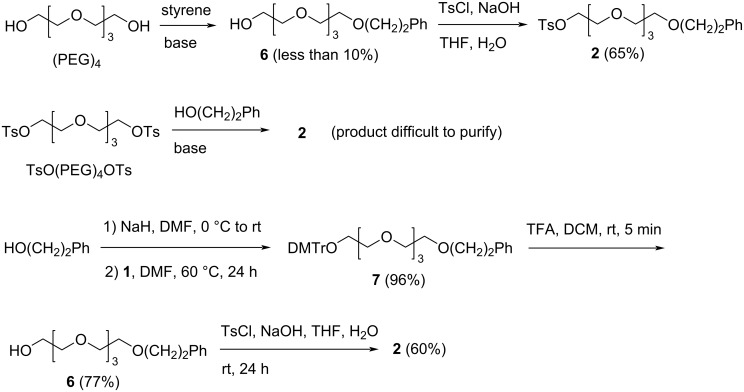
Synthesis of monomer **2**.

Using the phenethyl group for protection, the monomer **2** was chosen for the stepwise PEG synthesis. The simplest method for its synthesis would be to react (PEG)_4_, which is commercially available and inexpensive, with styrene to give **6** [32], and tosylation of **6** to give the monomer (Scheme 3). However, the reported conditions for the synthesis of **6** without using an expensive catalyst gave low yields. We did not test the conditions using the expensive catalyst that was used in the literature [32] due to cost considerations. Another method we tried was to react excess TsO(PEG)_4_OTs with 2-phenylethan-1-ol under basic conditions to give **2** (Scheme 3). However, separation of **2** from TsO(PEG)_4_OTs and Ph(CH_2_)_2_O(PEG)_4_O(CH_2_)_2_Ph required extensive chromatography. Thus, this method had been put aside. In our lab, we can produce **1** in large quantities without any chromatography [25], and therefore, we decided to use a route for the synthesis of **2** using **1** as the starting material. As shown in Scheme 3, 2-phenylethan-1-ol was reacted with **1** under basic conditions to give **7**. Removal of the DMTr group of **7** under acidic conditions gave **6**, which was tosylated to give **2**. This route is longer than the other two, but the products of all the steps are easy to purify, and it is our preferred route.

**Scheme 4:**
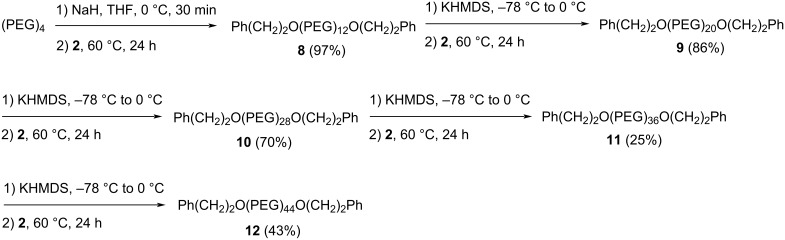
PEG synthesis using the one-pot PEG elongation approach.

With the monomer **2** in hand, the stepwise synthesis of monodisperse PEG using the one-pot elongation approach was investigated using the route in Scheme 4. The commercially available and inexpensive (PEG)_4_ was deprotonated with excess NaH and reacted with monomer **2**. This gives the (PEG)_12_ derivative **8**. The next reactions can elegantly show the convenience of the one-pot PEG elongation approach. The phenethyl group in **8** was removed with KHMDS and the intermediate alkoxide was reacted directly with **2** in one pot to give the (PEG)_20_ derivative **9**. The same procedure was simply repeated to give PEG derivatives Ph(CH_2_)_2_O(PEG)_28_O(CH_2_)_2_Ph (**10**), Ph(CH_2_)_2_O(PEG)_36_O(CH_2_)_2_Ph (**11**), and Ph(CH_2_)_2_O(PEG)_44_O(CH_2_)_2_Ph (**12**). In the PEG elongation process, we used excess KHMDS (2.5 equiv) for the deprotection to overcome inadvertent moisture. To prevent the excess base from deprotecting the phenethyl groups in the monomer, before adding the monomer, the reaction mixture was cooled to −78 °C, and then the monomer solution was added and the reaction mixture was warmed to room temperature slowly before heating to 60 °C. The careful manipulation of the temperature allowed the excess base to be selectively consumed via β-elimination of the tosylate of the monomer instead of removing its protecting group. As noted earlier, the side product of β-elimination of the tosylate does not affect the reaction while the side product of premature deprotection of the monomer would cause problems. The need of temperature manipulation may be regarded as a drawback of the one-pot PEG synthesis approach. However, it is reminded that in an industry setting where the reaction is performed at multiple gram or kilogram scales and the relative molar quantities of KHMDS and PEG starting materials can be controlled with high accuracy, cooling to low temperature is not needed. The yields of the one-pot PEG elongation reactions were not optimized. They ranged from 25% to 86%. We believe that the yields can be improved by more careful reaction workup and product purification, which is especially true for long PEG synthesis when the relatively hydrophobic phenethyl groups in the molecules are less capable to curtail the hydrophilicity of PEG moiety and to bring the product to the organic phase during aqueous workup. We also believe that the one-pot approach can be readily adopted for the synthesis of PEGs longer than (PEG)_44_. Two facts support this speculation. One is that PEG depolymerization did not appear to be a significant problem according to MS (Supporting Information File 1) [33]. The other is that according to TLC (Supporting Information File 1), the PEG products we made were not difficult to purify, and it is reasonable to predict that PEGs significantly longer than the ones we made will behave similarly. In addition, PEGs are soluble in solvents such as water, toluene, DCM and many other solvents but not soluble in diethyl ether and hexanes. This solubility pattern is very different from most other organic compounds including the side products of the PEG elongation reaction, which mainly include styrene and *p*-toluenesulfonic acid. As a result, when the reaction is performed at large scales, there is a high probability that the product can be purified by methods such as partition, precipitation and crystallization.

The major advantage of using a base-labile protecting group such as the phenethyl group for stepwise monodisperse PEG synthesis is the reduction of the PEG elongation cycle from three steps in two pots to two steps in one pot. Using the new approach, there is no need to isolate and purify the intermediate product after the deprotection step. Because stepwise PEG synthesis requires to repeat the PEG elongation cycle multiple times, shortening each cycle from two pots to one pot can make PEG synthesis significantly more convenient, which can render monodisperse PEGs more affordable. In addition, the omission of the isolation and purification of an intermediate in each of the multiple PEG elongation cycles can significantly reduce the use of harmful organic solvents and other chemicals. In the literature, besides the DMTr group, other protecting groups including benzyl and silyl groups have also been used for PEG synthesis [15,19,21,24]. However, like the DMTr group, when they were used, all required two pots for each PEG elongation. Therefore, the base-labile group is not only a better choice than the DMTr group, but also a better choice than any known protecting groups.

**Scheme 5:**
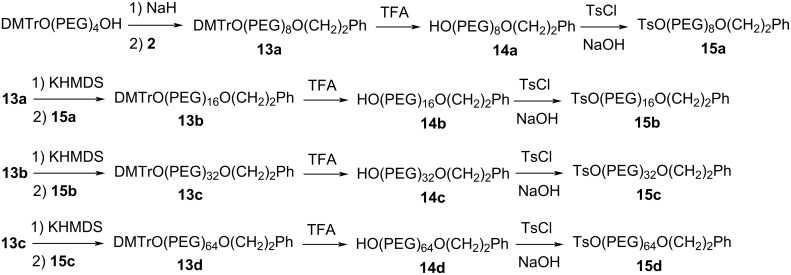
A proposed route for the synthesis of long and asymmetric PEGs using a base-labile protecting group.

There are a number of different routes for stepwise monodisperse PEG synthesis, which include unidirectional iterative coupling, bidirectional iterative coupling, chain doubling, and chain tripling [15]. The base-labile protecting strategy can be easily incorporated into all those routes, and the routes can be shortened significantly by carrying out deprotection and coupling in one pot. We demonstrated the convenience of the one-pot PEG elongation approach using bidirectional iterative coupling route (Scheme 3). This route has the advantage of using the same monomer in each elongation cycle. In addition, the length of the monomer is significantly shorter than that of the product, and therefore, excess monomer can be used to drive the PEG elongation reactions to completion because the excess monomer can be easily removed from the product. However, for the synthesis of PEGs longer than (PEG)_60_ or asymmetric PEGs, other routes such as that in Scheme 5 using two orthogonal protecting groups would be preferred. Such routes can double the length of the PEG in three easy steps and the PEG product is asymmetric.

It is noted that the use of base-labile protecting groups or linkers in organic synthesis involving carrying out reactions under less basic reactions and removing the protecting group or cleaving the linker under more basic conditions is not common. In contrast, the use of acid-labile protecting groups or linkers involving carrying out reactions under less acidic conditions and removing the protecting group or cleaving the linker under more acidic conditions is more frequently adopted. For example, in peptide synthesis, peptides with acid-labile side chain protections can be selectively cleaved from the acid-labile 2-chlorotrityl resin with dilute TFA [34]. In RNA synthesis, the acid-sensitive 2'-TOM protecting groups can survive the acidic conditions for removing the 5'-DMTr groups [35]. It is remarkable that in the one-pot PEG elongation approach, the base-labile protecting groups in the monomer and product are completely stable under the relatively harsh basic Williamson coupling conditions.

## Conclusion

In conclusion, a one-pot PEG elongation approach has been developed for the stepwise monodisperse PEG synthesis. By using a base-labile protecting group such as the phenethyl group instead of the commonly used groups such as the acid-labile DMTr group, each PEG elongation in stepwise PEG synthesis can be carried out in one pot instead of two pots. The deprotonation step is not needed using the new approach. In addition, due to the irreversibility of the reactions for their deprotection, the new protecting groups are also easier to remove. Our results showed that the PEG synthesis method is convenient to execute, and high yields of PEG products can be obtained. We expect that the one-pot PEG elongation approach will be helpful to make monodisperse PEGs more affordable and has a positive impact in areas such as bioconjugation and nanomedicine where monodisperse PEGs are needed.

## Supporting Information

File 1 Experimental details, images of TLC, and images of ^1^H and ^13^C NMR, and MS of new compounds.

## References

[1] Sellaturay P, Nasser S, Ewan P (2021). J Allergy Clin Immunol: Pract.

[2] d'Avanzo N, Celia C, Barone A, Carafa M, Di Marzio L, Santos H A, Fresta M (2020). Adv Ther (Weinheim, Ger).

[3] Soni J, Sahiba N, Sethiya A, Agarwal S (2020). J Mol Liq.

[4] Parray Z A, Hassan M I, Ahmad F, Islam A (2020). Polym Test.

[5] Constantinou C, Charalambous C, Kanakis D, Kolokotroni O, Constantinou A I (2021). Nutr Cancer.

[6] Panisello Rosello A, Teixeira da Silva R, Castro C, Bardallo R G, Calvo M, Folch-Puy E, Carbonell T, Palmeira C, Roselló Catafau J, Adam R (2020). Int J Mol Sci.

[7] Thompson M S, Vadala T P, Vadala M L, Lin Y, Riffle J S (2008). Polymer.

[8] Herzberger J, Niederer K, Pohlit H, Seiwert J, Worm M, Wurm F R, Frey H (2016). Chem Rev.

[9] Abd Ellah N H, Tawfeek H M, John J, Hetta H F (2019). Nanomedicine (London, U K).

[10] Tang W, Fan W, Lau J, Deng L, Shen Z, Chen X (2019). Chem Soc Rev.

[11] Beltrán-Gracia E, López-Camacho A, Higuera-Ciapara I, Velázquez-Fernández J B, Vallejo-Cardona A A (2019). Cancer Nanotechnol.

[12] Giorgi M E, Agusti R, de Lederkremer R M (2014). Beilstein J Org Chem.

[13] Pipe S W, Montgomery R R, Pratt K P, Lenting P J, Lillicrap D (2016). Blood.

[14] Li Y, Qiu X, Jiang Z-X (2015). Org Process Res Dev.

[15] French A C, Thompson A L, Davis B G (2009). Angew Chem, Int Ed.

[16] Wawro A M, Muraoka T, Kato M, Kinbara K (2016). Org Chem Front.

[17] Xia G, Li Y, Yang Z, Jiang Z-X (2015). Org Process Res Dev.

[18] Wawro A M, Muraoka T, Kinbara K (2016). Polym Chem.

[19] Muraoka T, Adachi K, Ui M, Kawasaki S, Sadhukhan N, Obara H, Tochio H, Shirakawa M, Kinbara K (2013). Angew Chem, Int Ed.

[20] Zhang H, Li X, Shi Q, Li Y, Xia G, Chen L, Yang Z, Jiang Z-X (2015). Angew Chem, Int Ed.

[21] Maranski K, Andreev Y G, Bruce P G (2014). Angew Chem, Int Ed.

[22] Wan Z, Li Y, Bo S, Gao M, Wang X, Zeng K, Tao X, Li X, Yang Z, Jiang Z-X (2016). Org Biomol Chem.

[23] Székely G, Schaepertoens M, Gaffney P R J, Livingston A G (2014). Chem – Eur J.

[24] Ahmed S A, Tanaka M (2006). J Org Chem.

[25] Khanal A, Fang S (2017). Chem – Eur J.

[26] Kinugasa S, Takatsu A, Nakanishi H, Nakahara H, Hattori S (1992). Macromolecules.

[27] Hay B A, Godugu K, Darwish N H E, Fujioka K, Sudha T, Karakus O O, Mousa S A (2021). J Med Chem.

[28] Halami B, Eriyagama D N A M, Chillar K, Nelson Z, Prehoda L, Yin Y, Lu B-Y, Otto B, Haggerty L, Fang S (2019). Tetrahedron Lett.

[29] Margot C, Rizzolio M, Schlosser M (1990). Tetrahedron.

[30] Margot C, Matsuda H, Schlosser M (1990). Tetrahedron.

[31] Fraser R R, Mansour T S, Savard S (1985). J Org Chem.

[32] Luo C, Bandar J S (2018). J Am Chem Soc.

[33] Boden N, Bushby R J, Clarkson S, Evans S D, Knowles P F, Marsh A (1997). Tetrahedron.

[34] Bray B L (2003). Nat Rev Drug Discovery.

[35] Jud L, Micura R (2017). Chem – Eur J.

