# Supplementary material for: Stepwise PEG synthesis featuring deprotection and coupling in one pot
Source: Beilstein J Org Chem. 2021 Dec 28;17:2976–82. doi: 10.3762/bjoc.17.207 (PMC8722398; doi:10.3762/bjoc.17.207)
Supplement: File 1 — Experimental details, images of TLC, and images of 1H and 13C NMR, and MS of new compounds. [file Beilstein_J_Org_Chem-17-2976-s001.pdf]

BEILSTEIN JOURNAL OF ORGANIC CHEMISTRY

Supporting Information

for

Stepwise PEG synthesis featuring deprotection and coupling in one pot

Logan Mikesell, Dhananjani N. A. M. Eriyagama, Yipeng Yin, Bao-Yuan Lu and Shiyue Fang

Beilstein J. Org. Chem. **2021,** *17,* 2976–2982. doi:10.3762/bjoc.17.207

Experimental details, images of TLC, and images of ^{1}H and ^{13}C NMR, and MS of new compounds

License and Terms: This is a supporting information file under the terms of the Creative Commons Attribution License (https://creativecommons.org/licenses/by/4.0). Please note that the reuse, redistribution and reproduction in particular requires that the author(s) and source are credited and that individual graphics may be subject to special legal provisions.
The license is subject to the *Beilstein Journal of Organic Chemistry* terms and conditions: (https://www.beilstein-journals.org/bjoc/terms)

Table of contents

Experimental details………………………………………………………………...……..S2

TLC images for screening base-labile protecting groups for PEG synthesis – Testing if the groups in **3a**–**l** can be removed under basic conditions……………………………………..………S6

TLC images for screening base-labile protecting groups for PEG synthesis – Testing stability of protecting groups under the basic Williamson ether formation conditions……….….………...S12

TLC, NMR and MS of new compounds……………………………………………………...S24

References………………………………………………………………………………S40

Experimental Details

General information: All compounds from commercial sources were used as received unless noted otherwise. THF was distilled over Na/benzophenone under nitrogen. Compounds **3d** [1], **3e** [2], **3g** [3], and **3j** [3] were synthesized following reported procedure. All reactions were carried out under nitrogen using oven-dried glassware. Thin layer chromatography (TLC) was performed using Sigma-Aldrich TLC plates, silica gel 60F-254 over glass support, 250 μm thickness. ^1H and ^{13}C NMR spectra were obtained on a Varian UNITY INOVA spectrometer at 400 and 100 MHz, respectively. Chemical shifts (δ) were reported in reference to solvent peaks (residue $CHCl_3$ at δ 7.24 ppm for ^1H and $CDCl_3$ at δ 77.00 ppm for ^{13}C). HRMS was obtained on a Thermo HR-Orbitrap Elite Mass Spectrometer. LRMS was obtained on a Thermo Finnigan LCQ Advantage Ion Trap Mass Spectrometer.

*Screening base-labile protecting groups for PEG synthesis – Testing if the groups in **3a-l** can be removed under basic conditions*: In an oven dried 25 mL flask, **3a**–**k** or **3l** (0.734 mmol, 1 equiv) was dissolved in THF (4 mL). The solution was cooled to −78 °C. KHMDS (1 M in THF, 1.468 mL, 1.468 mmol, 2 equiv) was added via a syringe. The reaction mixture was stirred while warming to 0 °C gradually. After 2 h, TLC analyses (see below) were carried out. All compounds **3a**–**l** were found to be consumed. Thus, the base-labile protecting groups in them meet the criterion of being labile under basic conditions required for PEG synthesis. Compound **3a** was also tested using the base *t*-BuOK/LDA and found consumed under the conditions [4,5].

Screening base-labile protecting groups for PEG synthesis – Testing stability of protecting groups under the basic Williamson ether formation conditions: Compounds DMTrO(PEG)$_4$OTs (**1**) [6] and MeO(PEG)$_4$OH (**4**) were dried over P_2O_5 in a desiccator under vacuum for 2 days. Compound **4** (41 mg, 0.201 mmol, 1 equiv) was dissolved in THF (200 μL) under nitrogen. The solution was cooled to −78 °C, and KHMDS (0.241 mL, 0.241 mmol, 1 M in THF, 1.2 equiv) was added dropwise via a syringe. After addition, the reaction flask was placed in an ice bath for ~30 min. The mixture was then cooled to −78 °C. The solution of **1** (195 mg, 0.301 mmol, 1.5 equiv) and **3a**–**k** or **3l** (0.301 mmol, 1.5 equiv) in THF (500 μL) was added via a cannula dropwise. The reaction mixture was warmed to rt gradually over ~3 h. After stirring at rt for ~30 min, the mixture was heated to 60 °C and stirred vigorously at the temperature for 24 h. TLC analyses (see below) were carried out to determine if the Williamson ether formation reaction could proceed to form product **5** without the consumption of compound **3a**–**k** or **3l**. All the compounds except **3h** were found to be able to survive the basic Williamson ether formation reaction conditions. Thus, the base-labile protecting groups in them (except **3h**) meet the criterion of being stable under basic coupling conditions required for PEG synthesis.

*DMTrO(PEG)$_4$O(CH$_2$)$_2$Ph (**7**)*: The suspension of NaH (60% in mineral oil, 716 mg, 17.9 mmol, 2.5 equiv.) in anhydrous DMF (25 mL) in a 2-neck round bottom flask under nitrogen was cooled on an ice bath. The solution of Ph(CH$_2$)$_2$OH (2.14 mL, 17.9 mmol, 2.5 equiv) in anhydrous DMF (15 mL) was added dropwise via a cannula over ~1 h. After addition, the reaction mixture was stirred at 0 °C for ~1 h. The ice bath was removed. This gave the solution of NaO(CH$_2$)$_2$Ph. Compound **1** (4.66 g; 7.17 mmol, 1 equiv), which had been dried over P_2O_5 under high vacuum overnight, was dissolved in anhydrous DMF (15 mL). The solution was added to the solution of

$NaO(CH_2)_2Ph$ dropwise via a cannula. After addition, the mixture was stirred vigorously at 60 °C for 24 h. After cooling to rt, the reaction was quenched with EtOH. DMF was removed on a rotary evaporator under high vacuum. The residue was partitioned between EtOAc (250 mL) and 5% K_2CO_3 (100 mL). The organic phase was washed with 5% K_2CO_3 (100 mL × 3), dried over anhydrous Na_2SO_4, and filtered. The filtrate was evaporated to dryness under reduced pressure and further dried under high vacuum. The residue was purified with flash chromatography (SiO_2, Et_3N/hexanes 1:9) to give compound **7** (4.02 g, 96%) as a yellow oil: TLC R_f = 0.3 (SiO_2, hexanes/EtOAc 3:1); ^1H NMR (400 MHz, $CDCl_3$) δ 7.49-7.47 (d, 2H), 7.37-7.35 (d, 4H), 7.29-7.18 (m, 8H), 6.83-6.80 (m, 4H), 2.76-2.69 (m, 8H), 3.74 (s, 6H), 3.68-3.59 (m, 16H), 3.25-3.23 (t, 2H), 2.91-2.87 (t, 2H); ^{13}C NMR (100 MHz, $CDCl_3$) δ 158.5, 145.3, 139.1, 136.5, 130.3, 129.1, 128.5, 128.4, 127.9, 126.8, 126.3, 113.2, 86.2, 72.6, 71.0, 70.5, 63.5, 55.5, 36.6; HRMS (ESI) calcd for $C_{37}H_{43}O_7Na$ $[M+Na]^+$ 623.2985, found 623.2971.

*$Ph(CH_2)_2O(PEG)_4$ (**6**)*: Compound **7** (2.17 g, 3.62 mmol, 1 equiv.) was dissolved in dry DCM (10 mL). To the solution was added TFA (433 µL, 3.62 mmol, 1 equiv). The reaction mixture was stirred vigorously. After ~5 min, TLC indicated that compound **7** was consumed. The reaction was quenched with solid NaOH and a small volume of water until pH ~9. The mixture was then partitioned between DCM (total about 200 mL) and brine (75 mL). The aqueous phase was washed with DCM (100 mL × 3). The combined organic phase was dried over anhydrous Na_2SO_4, and filtered. The filtrate was evaporated to dryness, and the residue was purified with flash chromatography (SiO_2, EtOAc) to give compound **6** (568 mg, 77%) as a yellow oil: TLC R_f = 0.10 (SiO_2, hexanes/EtOAc 1:3); ^1H NMR (400 MHz, $CDCl_3$) δ 7.25-7.10 (m, 5H), 3.67-3.64 (t, 2H), 3.62-3.53 (m, 16H), 2.87-2.83 (t, 2H); ^{13}C NMR (100 MHz, $CDCl_3$) δ 138.9, 129.1, 128.5, 126.3, 72.8, 70.8, 70.5, 91.9, 36.5; HRMS (ESI) calcd for $C_{16}H_{26}O_5H$ $[M+H]^+$ 299.1859, found 299.1847; $C_{16}H_{26}O_5Na$ $[M+Na]^+$ 321.1678, found 321.1662.

*$Ph(CH_2)_2O(PEG)_4OTs$ (**2**)*: The compound was synthesized using a reported procedure with modifications [7]. The solutions of **6** (9.22 g, 46.5 mmol, 1 equiv) in THF (50 mL) and NaOH powder (22.3 g, 557 mmol, 12 equiv) in water (50 mL) were combined and stirred at 0 °C for 5 min. The solution of TsCl (26.5 g, 139.5 mmol, 3 equiv) in THF (50 mL, note that it is important to keep the ratio of total THF and water at around 2:1 v/v) was added dropwise over 10 min while the reaction mixture was stirred at 0 °C. After addition, stirring was continued while the temperature was raised to rt gradually. The progress of the reaction was monitored by TLC, and complete reaction was observed within 24 h. The mixture was partitioned between 5% Na_2CO_3 (300 mL) and EtOAc (500 mL). The aqueous phase was extracted with EtOAc (200 mL × 3). The combined organic phase was dried over anhydrous Na_2SO_4 and filtered. Volatiles were removed under reduced pressure, and the residue was further dried under vacuum from an oil pump. Compound **2** (12.7 g, 60%) was obtained as a colorless oil after flash chromatography purification (SiO_2, hexanes/EtOAc 1:0 to 2:1): TLC R_f = 0.30 (SiO_2, hexanes/EtOAc 1:1); ^1H NMR (400 MHz, $CDCl_3$) δ 7.78-7.76 (d, 2H), 7.32-7.30 (d, 2H), 7.27-7.16 (m, 5H), 4.14-4.12 (t, 2H), 3.68-3.59 (m, 16H), 2.89-2.86 (t, 2H), 2.42 (s, 3H); ^{13}C NMR (100 MHz, $CDCl_3$) δ 144.9, 139.0, 133.2, 129.9, 129.1, 128.5,128.1, 126.3, 72.5, 70.9, 70.8, 70.7, 70.5, 69.5, 68.9, 36.5, 21.9; HRMS (ESI) calcd for $C_{23}H_{31}O_7SH$ $[M+H]^+$ 453.1942, found 453.1953; $C_{23}H_{31}O_7SNH_4$ $[M+NH_4]^+$ 470.2207, found 470.2216; $C_{23}H_{31}O_7SNa$ $[M+Na]^+$ 474.1761, found 475.1775.

*$CH_2)_2O(PEG)_{12}O(CH_2)_2Ph$ (**8**)*: Compound **2** (2.19 g, 4.83 mmol, 2.5 equiv.) was dried over P_2O_5 under vacuum in a desiccator overnight. A suspension of NaH (60% in mineral oil, 193 mg, 4.83 mmol, 2.5 equiv) in dry THF (5 mL) under nitrogen was cooled on an ice bath. The solution of $(PEG)_4$ (333 µL, 1.93 mmol, 1 equiv) in dry THF (10 mL) was added via a cannula dropwise over ~20 min. After addition, the reaction was allowed to proceed for ~30 min. The ice bath was removed, and compound **2** in THF (10 mL) was added via a cannula dropwise over ~10 min. After addition, the mixture was stirred vigorously at 60 °C for 24 h. The reaction was quenched with EtOH. THF was removed under reduced pressure. The residue was partitioned between DCM (100 mL) and saturated NH_4Cl (50 mL). The aqueous phase was washed with DCM (100 mL × 3). The combined organic phase was dried over anhydrous Na_2SO_4 and filtered. The filtrate was evaporated to dryness, and compound **8** was purified with flash chromatography (SiO_2, EtOAc/MeOH 100:0 to 100:3) to give a colorless oil (1.4 g, 97%): TLC R_f = 0.50 (SiO_2, DCM/Et_2O/MeOH 5:1:1); ^{1}H NMR (400 MHz, $CDCl_3$) δ 7.24-7.712 (m, 10H), 3.64-3.55 (m 51H), 2.87-2.83 (t, 4H); ^{13}C NMR (100 MHz, $CDCl_3$) δ 133.9, 129.02, 128.44, 126.28, 72.52, 70.80, 70.90, 36.54 HRMS (ESI) calcd for $C_{40}H_{66}O_{13}Na$ $[M+Na]^+$ 777.4401, found 777.4436; $C_{40}H_{66}O_{13}Na_2$ $[M+2Na]^{2+}$ 400.2150, found 400.2112.

*$Ph(CH_2)_2O(PEG)_{20}O(CH_2)_2Ph$ (**9**)*: Compounds **2** and **8** were dried over P_2O_5 in a desiccator under vacuum for 2 days. Compound **8** (1.3 g, 1.8 mmol, 1 equiv) was dissolved in dry THF (5 mL) under nitrogen. The solution was cooled to −78 °C, and KHMDS (4.6 mL, 1 M in THF, 2.5 equiv) was added dropwise via a syringe. After addition, the reaction flask was placed in an ice bath for ~3 h. TLC analysis indicated that both **8** and $Ph(CH_2)_2O(PEG)_{12}$ were not in the reaction mixture. The mixture was then cooled to −78 °C for ~10 min, and the solution of **2** (3.8 g, 8.3 mmol, 4.5 equiv.) in THF (10 mL) was added dropwise via a cannula over ~10 min. The reaction mixture was allowed to warm up to room temperature gradually over a period of ~3 h. After stirring at room temperature for ~30 min, the mixture was heated to 60 °C and stirred vigorously at the temperature for 24 h. THF was removed under reduced pressure. The residue was partitioned between DCM (100 mL) and saturated NH_4Cl (20 mL). The aqueous phase was washed with DCM (100 mL × 3). The combined organic phase was dried over anhydrous Na_2SO_4 and filtered. Flash chromatography (SiO_2, EtOAc to DCM/Et_2O/MeOH 100:8:4) gave compound **9** (1.765 g, 86%) as a yellow waxy solid: TLC R_f = 0.40 (SiO_2, DCM/Et_2O/MeOH 10:1:1); ^{1}H NMR (400 MHz, $CDCl_3$) δ 7.28-7.15 (m, 10H), 3.67-3.57 (m 81H), 2.90-2.88 (t, 4H); ^{13}C NMR (100 MHz, $CDCl_3$) δ 138.97, 129.08, 128.50, 126.34, 72.60, 70.80, 70.50, 36.55; HRMS (ESI) calcd for $C_{56}H_{98}O_{21}Na$ $[M+Na]^+$ 1129.6499, found 1129.6533; $C_{56}H_{98}O_{21}H_2$ $[M+2H]^{2+}$ 554.3379, found 554.3390. Compound **9** was also synthesized using tBuOK/LDA instead of KHMDS as the base under otherwise identical conditions. Similar yields were obtained.

*$Ph(CH_2)_2O(PEG)_{28}O(CH_2)_2Ph$ (**10**)*: Synthesized using the procedure for the synthesis of **9**. Compound **9** (1.77 g, 1.59 mmol, 1 equiv) in THF (10 mL), KHMDS (3.39 mL, 1 M in THF, 2.2 equiv), and **2** (3.24 g, 7.15 mmol, 4.5 equiv) in THF (10 mL) gave the crude product, which was subjected to aqueous workup and chromatography purification as describe for **9**. Compound **10** (1.6 g, 70%) was obtained as a yellow waxy solid: TLC R_f = 0.40 (SiO_2, DCM/Et_2O/MeOH 10:1:1); ^{1}H NMR (400 MHz, $CDCl_3$) δ 7.27-7.15 (m, 10H), 3.67-3.56 (m 116H), 2.89-2.85 (t, 4H); ^{13}C NMR (100 MHz, $CDCl_3$) δ 139.02, 129.04, 128.46, 126.30, 72.54, 70.80, 36.56; HRMS (ESI)

calcd for $C_{74}H_{134}O_{30}Na$ $[M+Na]^+$ 1481.8596, found 1481.8571; $C_{74}H_{134}O_{30}Na_2$ $[M+2Na]^{2+}$ 752.4247, found 752.4247; $C_{74}H_{134}O_{30}H_3$ $[M+3H]^{3+}$ 487.2977, found 487.2971.

*$Ph(CH_2)_2O(PEG)_{36}O(CH_2)_2Ph$ (**11**)*: Synthesized using the procedure for the synthesis of **9**. Compound **10** (1.375 g, 0.942 mmol, 1 equiv) in THF (10 mL), KHMDS (2.4 mL, 1 M in THF, 2.5 equiv), and 2 (1.7 g, 3.8 mmol, 4 equiv) in THF (10 mL) gave the crude product, which was subjected to aqueous workup and chromatography purification as describe for **9**. Compound **11** (436 mg, 25%) was obtained as a yellow waxy solid: TLC R_f = 0.40 (SiO_2, DCM/Et_2O/MeOH 10:1:1); ^1H NMR (400 MHz, $CDCl_3$) δ 7.25-7.15 (m, 10H), 3.65-3.59 (m 148H), 2.87-2.83 (t, 4H); ^{13}C NMR (100 MHz, $CDCl_3$) δ 139.00, 129.02, 128.44, 126.29, 72.53, 70.79, 70.49, 36.54; HRMS (ESI) calcd for $C_{88}H_{162}O_{37}N_2H_8$ $[M+2NH_4]^{2+}$ 923.5742, found 923.5701; $C_{88}H_{162}O_{37}N_3H_{12}$ $[M+3NH_4]^{3+}$ 621.7276, found 621.7269.

*$Ph(CH_2)_2O(PEG)_{44}O(CH_2)_2Ph$ (**12**)*: Synthesized using the procedure for the synthesis of **9**. Compound **11** (386 mg, 0.241 mmol, 1 equiv) in THF (10 mL), KHMDS (0.532 mL, 1 M in THF, 2.5 equiv), and **2** (436 mg, 0.964 mmol, 4 equiv) in THF (10 mL) gave the crude product, which was subjected to aqueous workup and chromatography purification as describe for **9**. Compound **12** (199 mg, 43%) was obtained as a yellow waxy solid: TLC, R_f = 0.50 (SiO_2, DCM/Et_2O/MeOH 6:1:1); ^1H NMR (400 MHz, $CDCl_3$) δ 7.23-7.13 (m, 10H), 3.76-3.38 (m 179H), 2.85-2.81 (t, 4H); ^{13}C NMR (100 MHz, $CDCl_3$) δ 138.95, 129.02, 128.44, 126.29, 72.53, 70.77, 70.47, 36.52; HRMS (ESI) calcd for $C_{104}H_{194}O_{45}N_2H_8$ $[M+2NH_4]^{2+}$ 1099.6790, found 1099.6711; $C_{104}H_{194}O_{45}N_3H_{12}$ $[M+3NH_4]^{3+}$ 739.1308, found 739.1266; $C_{104}H_{194}O_{45}N_4H_{16}$ $[M+4NH_4]^{4+}$ 558.8663, found 558.8548.

TLC images for screening base-labile protecting groups for PEG synthesis – Testing if the groups in 3a–l can be removed under basic conditions

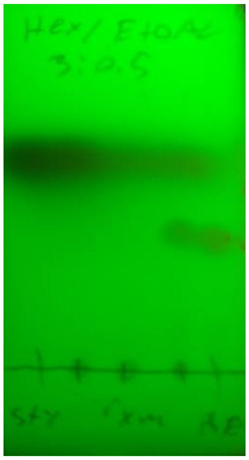

Figure S1. TLC for testing if -(CH_2)$_2$Ph group can be deprotected using KHMDS. Left lane: styrene; middle lane: reaction mixture; right lane: Ph(CH_2)$_2$OMe (**3a**); 2nd and 4th lanes: co-spot of materials spotted on their adjacent lanes. Eluent: hexanes/EtOAc 3:0.5. The TLC indicates that **3a** was consumed and styrene was formed.

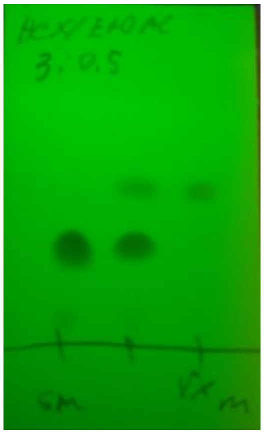

Figure S2. TLC for testing if -(CH_2)$_2$Ph(4-OMe) group can be deprotected using KHMDS. Left lane: MeO(CH_2)$_2$Ph(4-OMe) (**3b**); middle lane: co-spot of materials on the left and right lanes; right lane: reaction mixture. Eluent: hexanes/EtOAc 3:0.5. The TLC indicates that **3b** was consumed.

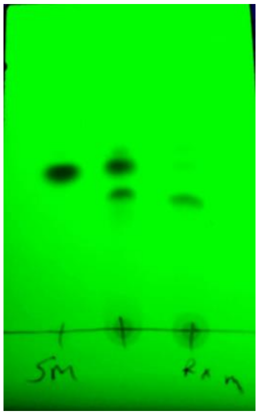

Figure S3. TLC for testing if -$(CH_2)_2Ph(4\text{-}NO_2)$ group can be deprotected by KHMDS. Left lane: $MeO(CH_2)_2Ph(4\text{-}NO_2)$ (**3c**); middle lane: co-spot of materials on left and right lanes; right lane: reaction mixture. Eluent: hexanes/EtOAc 3:0.5. The TLC indicates that **3c** was consumed.

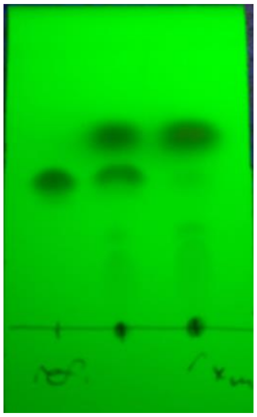

Figure S4. TLC for testing if -$(CH_2)_2Ph(3\text{-}F)$ group can be deprotected by KHMDS. Left lane: $MeO(CH_2)_2Ph(3\text{-}F)$ (**3d**); middle lane: co-spot of materials on left and right lanes; right lane: reaction mixture. Eluent: hexanes/EtOAc 3:1. The TLC indicates that **3d** was consumed.

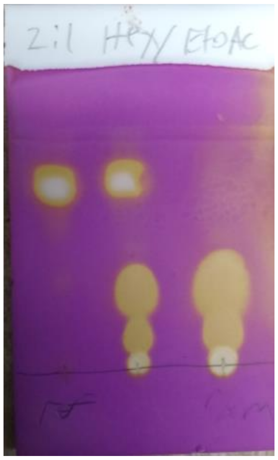

Figure S5. TLC for testing if the 2-(furan-2-yl)ethyl group can be deprotected by KHMDS. Left lane: 2-(2-methoxyethyl)furan (**3e**); middle lane: co-spot of materials on left and right lanes; right lane: reaction mixture. Eluent: hexanes/EtOAc 2:1. The TLC indicates that **3e** was consumed.

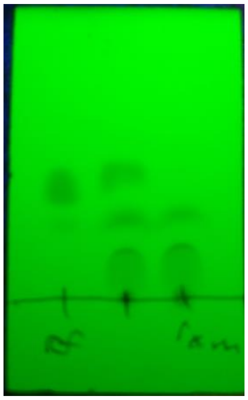

Figure S6. TLC for testing if -$(CH_2)_2CH{=}CH_2$ group can be deprotected by KHMDS. Left lane: $BnO(CH_2)_2CH{=}CH_2$ (**3f**); middle lane: co-spot of materials on left and right lanes; right lane: reaction mixture. Eluent: hexanes/EtOAc 9:1. The TLC indicates that **3f** was consumed.

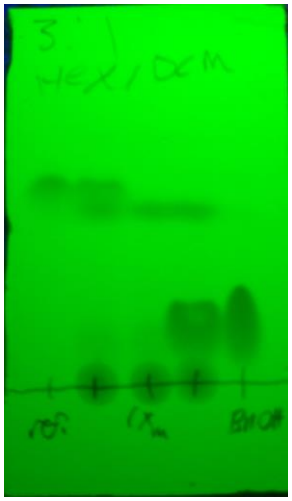

Figure S7. TLC for testing if -$(CH_2)_2C{\equiv}CMe$ group can be deprotected by KHMDS. Left lane: $BnO(CH_2)_2C{\equiv}CMe$ (**3g**); middle lane, reaction mixture; right lane: BnOH; 2nd and 4th lanes, co-spot of materials spotted on their adjacent lanes. Eluent: hexanes/DCM 3:1. The TLC indicates that **3g** was consumed.

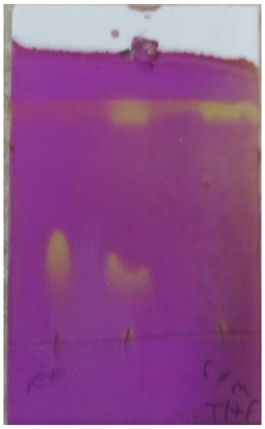

Figure S8. TLC for testing if -$(CH_2)_2C(=O)NMe_2$ group can be deprotected by KHMDS. Left lane: $MeO(CH_2)_2C(=O)NMe_2$ (**3h**); middle lane: co-spot of materials on left and right lanes; right lane: reaction mixture. Eluent: EtOAc. The TLC indicates that **3h** was consumed.

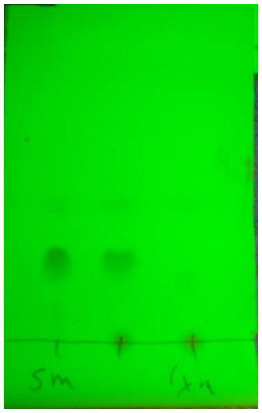

Figure S9. TLC for testing if -$(CH_2)_2CN$ can be deprotected by KHMDS. Left lane: $BnO(CH_2)_2CN$ (**3i**); middle lane: co-spot of materials on left and right lanes; right lane: reaction mixture. Eluent: hexanes/EtOAc 3:0.5. The TLC indicates that **3i** was consumed.

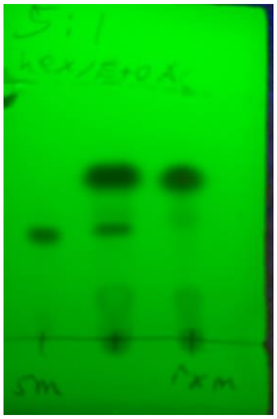

Figure S10. TLC for testing if -$CH_2CH(SCH_2)_2CH_2$ group can be deprotected by KHMDS. Left lane: $BnOCH_2CH(SCH_2)_2CH_2$ (**3j**); middle lane: co-spot of materials on the left and right lanes; right lane: reaction mixture. Eluent: hexanes/EtOAc 5:1. The TLC indicates that **3j** was consumed.

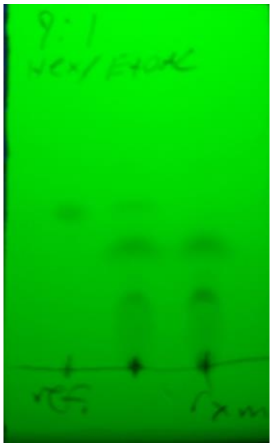

Figure S11. TLC for testing if $-CH_2CH{=}CHMe$ group can be deprotected by KHMDS. Left lane: $BnOCH_2CH{=}CHMe$ (**3k**); middle lane: co-spot of materials on left and right lanes; right lane: reaction mixture. Eluent: hexanes/EtOAc 9:1. The TLC indicates that **3k** was consumed.

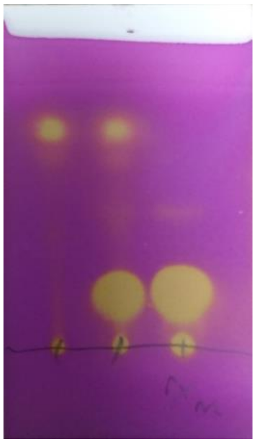

Figure S12. TLC for testing if $-CH_2C{\equiv}CMe$ group can be deprotected by KHMDS. Left lane: $EtOCH_2C{\equiv}CMe$ (**3l**); middle lane: co-spot of materials on left and right lanes; right lane: reaction mixture. Eluent: hexanes/EtOAc 5:1. The TLC indicates that **3l** was consumed.

TLC images for screening base-labile protecting groups for PEG synthesis – Testing stability of protecting groups under the basic Williamson ether formation conditions

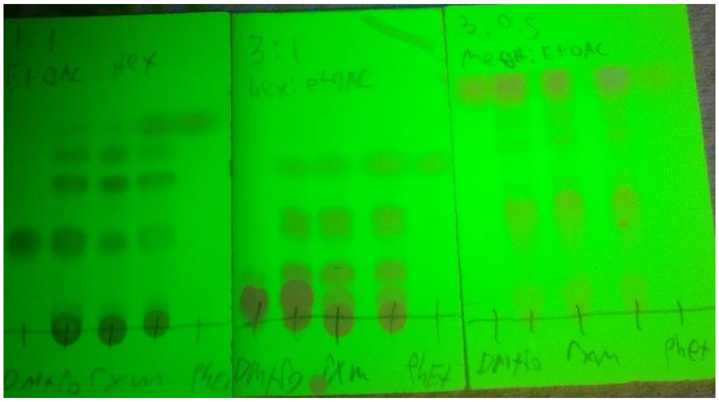

Figure S13. TLC for testing the stability of -$(CH_2)_2$Ph group under Williamson ether formation conditions. For all three TLC: left lane, DMTrO(PEG)$_4$OTs (**1**); middle lane, reaction mixture; right lane, Ph$(CH_2)_2$OMe (**3a**); 2nd and 4th lanes, co-spot of materials spotted on their adjacent lanes. Eluent: left TLC, EtOAc/hexanes 1:1; middle TLC, EtOAc/hexanes 1:3; right TLC, EtOAc/MeOH 3:0.5. Left and middle TLC indicate that **3a** was not consumed. Right TLC indicates that DMTrO(PEG)$_8$OMe (**5**), which has a R_f of 0.40 and identified with ESI MS, was formed.

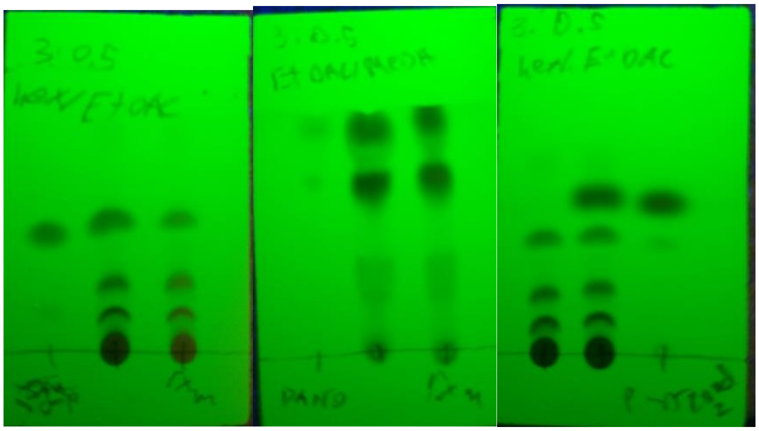

Figure S14. TLC for testing the stability of -$(CH_2)_2$Ph(4-OMe) group under Williamson ether formation conditions. Left TLC: left lane, MeO$(CH_2)_2$Ph(4-OMe) (**3b**); middle lane, co-spot of materials on the left and right lanes; right lane, reaction mixture. The TLC shows that **3b** was not consumed. Middle TLC: left lane, DMTrO$(PEG)_8$OMe (**5**); middle lane, co-spot of materials on the left and right lanes; right lane, reaction mixture. The TLC shows that product **5** was formed. Right TLC: left lane, reaction mixture; middle lane, co-spot of materials on the left and right lanes; righ lane, reaction mixture of the β-elimination reaction of **3b**. The TLC shows that the β-elimination product of **3b** was not formed. Eluent: left TLC, hexanes/EtOAc 3:0.5; middle TLC, EtOAc/MeOH 3:0.5; right TLC, hexanes/EtOAc 3:0.5.

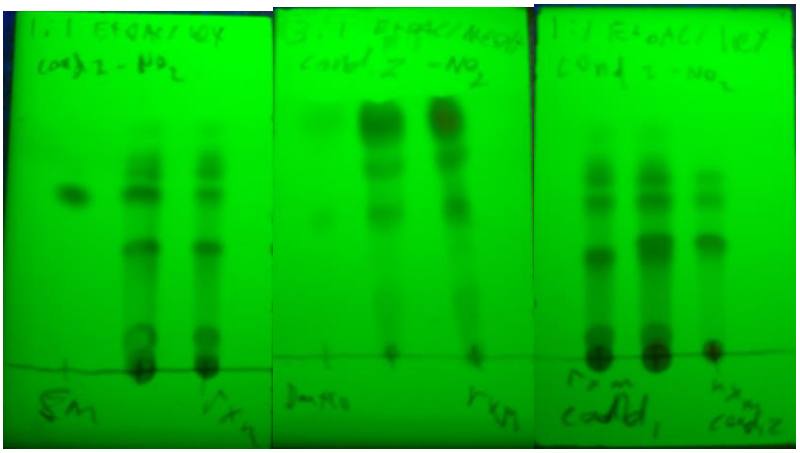

Figure S15. TLC for testing the stability of -$(CH_2)_2Ph(4\text{-}NO_2)$ group under Williamson ether formation conditions. Left TLC: left lane, $MeO(CH_2)_2Ph(4\text{-}NO_2)$ (**3c**); middle lane, co-spot of materials on the left and right lanes; right lane, reaction mixture. The TLC shows that **3c** was not consumed. Middle TLC: left lane, DMTrO(PEG)$_8$OMe (**5**); middle lane, co-spot of material on the left and right lanes; right lane, reaction mixture. The TLC shows that **5** was formed. Right TLC: left lane, reaction mixture; middle lane, co-spot of materials on the left and right lanes; right lane, reaction mixture of the β-elimination reaction of **3c**. The TLC shows that the β-elimination product of **3c** was not formed. Eluent: left TLC, hexanes/EtOAc 1:1; middle TLC, EtOAc/MeOH 3:1; right TLC, hexanes/EtOAc 1:1.

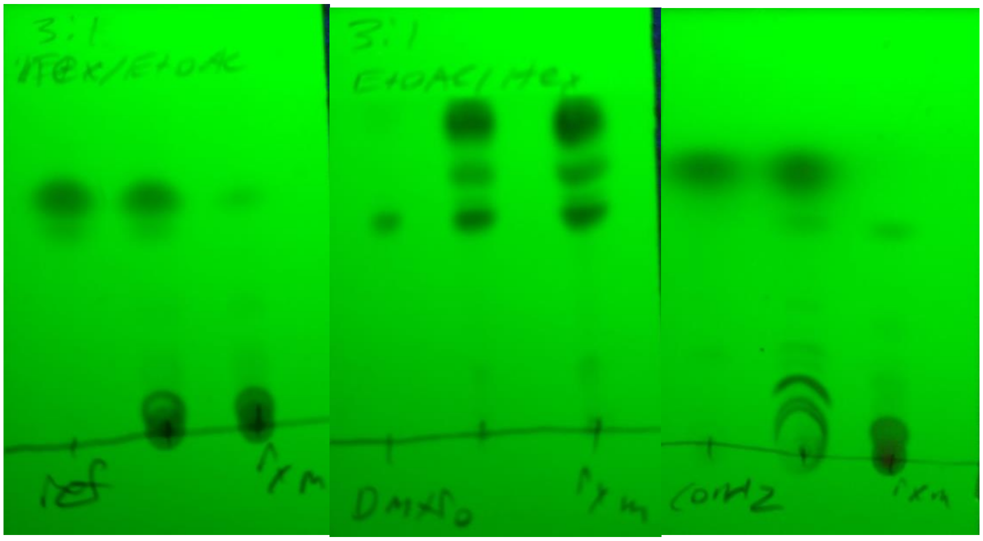

Figure S16. TLC for testing the stability of -$(CH_2)_2Ph$(3-F) group under Williamson ether formation conditions. Left TLC: left lane, $MeO(CH_2)_2Ph$(3-F) (**3d**); middle lane, co-spot of materials on the left and right lanes; right lane, reaction mixture. The TLC shows that **3d** was not consumed. Middle TLC: left lane, DMTrO$(PEG)_8$OMe (**5**); middle lane, co-spot of material on the left and right lanes; right lane, reaction mixture. The TLC shows that **5** was formed. Right TLC: left lane, reaction mixture of the β-elimination reaction of **3d**; middle lane, co-spot of materials on the left and right lanes; right lane, reaction mixture. The TLC shows that the β-elimination product of **3d** was not formed. Eluent: left TLC, hexanes/EtOAc 3:1; middle TLC, EtOAc/MeOH 3:1; right TLC, hexanes/EtOAc 3:1.

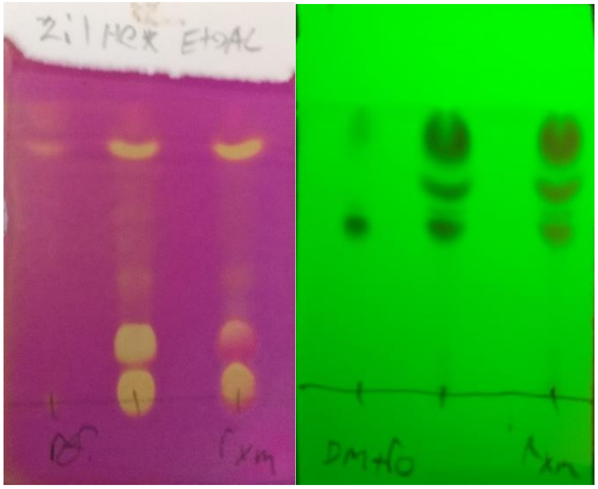

Figure S17. TLC for testing the stability of the 2-(furan-2-yl)ethyl group under Williamson ether formation conditions. Left TLC: left lane, 2-(2-methoxyethyl)furan (**3e**); middle lane, co-spot of materials on the left and right lanes; right lane, reaction mixture. The TLC shows that the **3e** was not consumed. Right TLC: left lane, DMTrO(PEG)$_8$OMe (**5**); middle lane, co-spot of material on the left and right lanes; right lane, reaction mixture. The TLC shows that **5** was formed. Eluent: left TLC, hexanes/EtOAc 2:1; right TLC, EtOAc/MeOH 3:1.

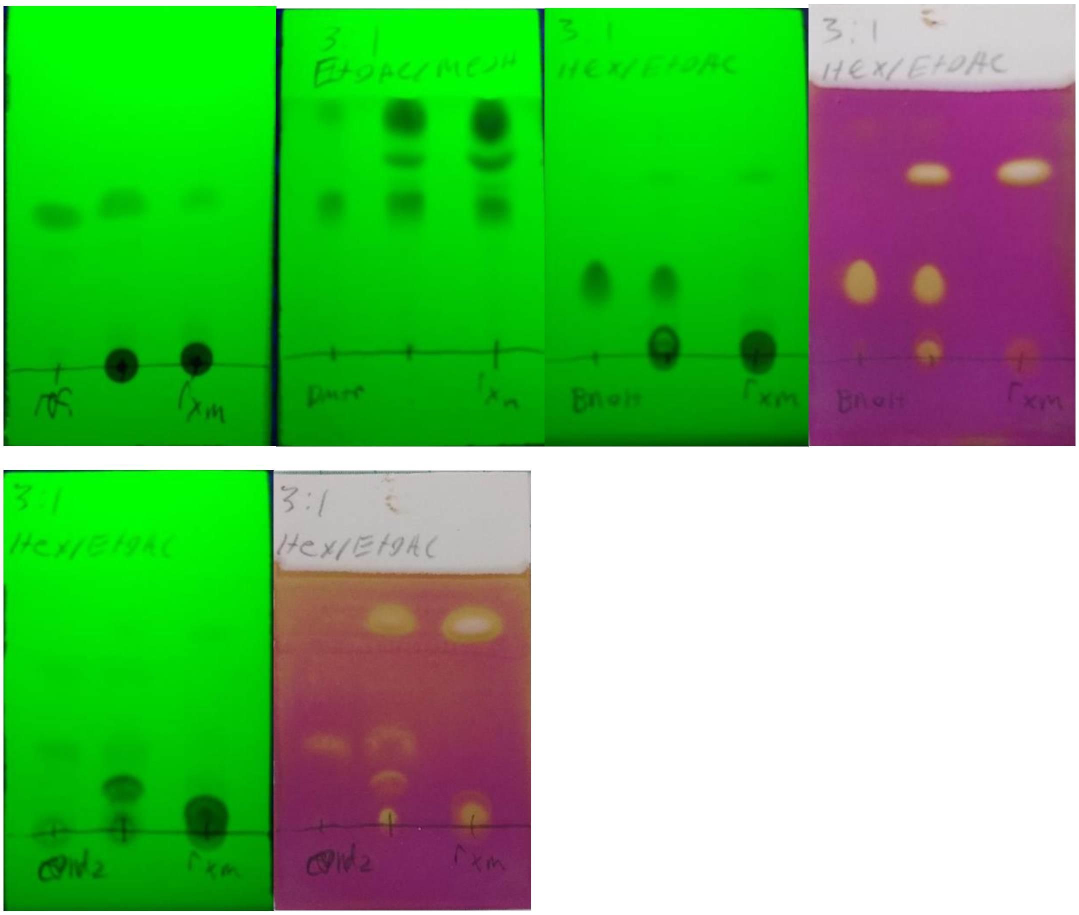

Figure S18. TLC for testing the stability of -$(CH_2)_2CH{=}CH_2$ group under Williamson ether formation conditions. Reading from left to right, first TLC: left lane, $BnO(CH_2)_2CH{=}CH_2$ (**3f**); middle lane, co-spot of materials on the left and right lanes; right lane, reaction mixture. The TLC shows that **3f** was not consumed. Second TLC: left lane, DMTrO(PEG)$_8$OMe (**5**); middle lane, co-spot of material on the left and right lanes; right lane, reaction mixture. The TLC shows that **5** was formed. Third TLC and fourth TLC ($KMnO_4$ stain): left lane, BnOH; middle lane, co-spot of materials on the left and right lanes; right lane, reaction mixture. The two TLCs show that β-elimination product was not formed. Fifth TLC and sixth TLC ($KMnO_4$ stain): left lane, reaction mixture of the β-elimination reaction of **3f**; middle lane, co-spot of materials on the left and right lanes; right lane, reaction mixture. The two TLC show that the β-elimination product of **3f** was not formed. Eluent: first TLC, hexanes/EtOAc 9:1; second TLC, EtOAc/MeOH 3:1; third and fourth TLCs, hexanes/EtOAc 3:1; fifth and sixth TLCs, hexanes/EtOAc 3:1.

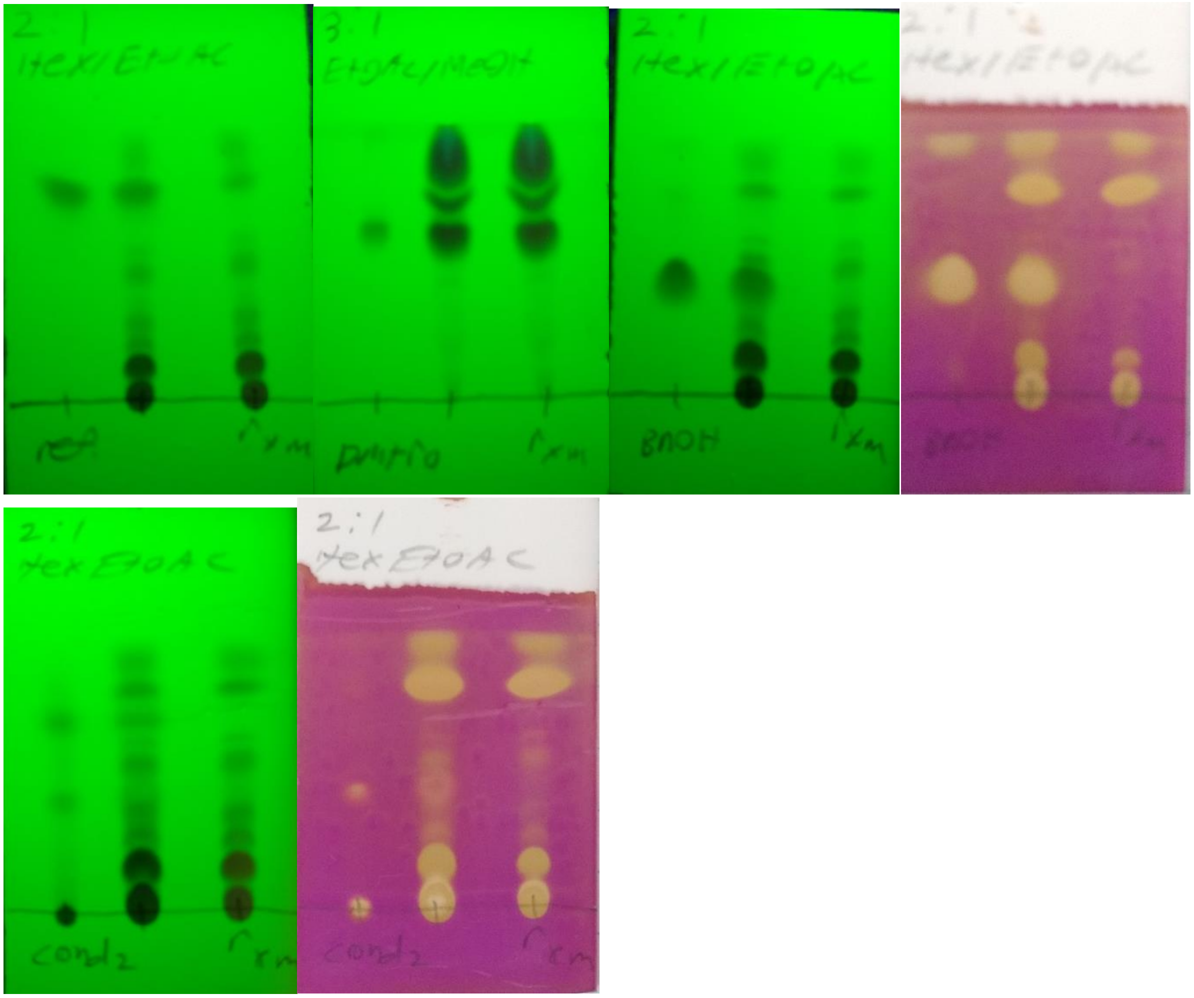

Figure S19. TLC for testing the stability of -$(CH_2)_2C{\equiv}CMe$ group under Williamson ether formation conditions. Reading from left to right, first TLC: left lane, $BnO(CH_2)_2C{\equiv}CMe$ (**3g**); middle lane, co-spot of materials on the left and right lanes; right lane, reaction mixture. The TLC shows that **3g** was not consumed. Second TLC: left lane, $DMTrO(PEG)_8OMe$ (**5**); middle lane, co-spot of material on the left and right lanes; right lane, reaction mixture. The TLC shows that **5** was formed. Third TLC and fourth TLC ($KMnO_4$ stain): left lane, BnOH; middle lane, co-spot of materials on the left and right lanes; right lane, reaction mixture. The TLCs show that β-elimination product was not formed. Fifth TLC and sixth TLC ($KMnO_4$ stain): left lane, reaction mixture of the β-elimination reaction of **3g**; middle lane, co-spot of materials on the left and right lanes; right lane, reaction mixture. The TLCs show that the β-elimination product of **3g** was not formed. Eluent: first TLC, hexanes/EtOAc 3:1; second TLC, EtOAc/MeOH 3:1; third and fourth TLCs, hexanes/EtOAc 3:1; fifth and sixth TLCs, hexanes/EtOAc 3:1.

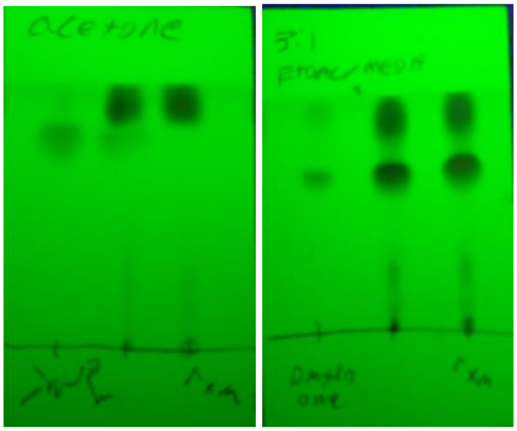

Figure S20. TLC for testing the stability of -$(CH_2)_2C(=O)NMe_2$ group under Williamson ether formation conditions. Left TLC: left lane, $MeO(CH_2)_2C(=O)NMe_2$ (**3h**); middle lane, co-spot of materials on the left and right lanes; right lane, reaction mixture. The TLC shows that the **3h** was consumed. Right TLC: left lane, DMTrO(PEG)$_8$OMe (**5**); middle lane, co-spot of material on the left and right lanes; right lane, reaction mixture. The TLC shows that **5** was formed. Eluent: left TLC, acetone; right TLC, EtOAc/MeOH 3:1.

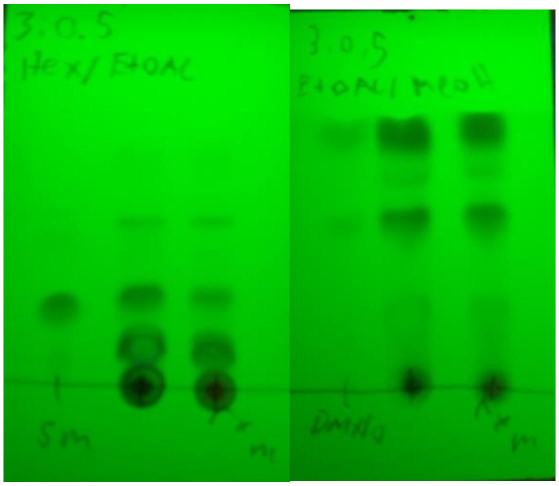

Figure S21. TLC for testing the stability of -$(CH_2)_2CN$ group under Williamson ether formation conditions. Left TLC: left lane, $BnO(CH_2)_2CN$ (**3i**); middle lane, co-spot of materials on the left and right lanes; right lane, reaction mixture. The TLC shows that the **3i** was not consumed. Right TLC: left lane, DMTrO(PEG)$_8$OMe (**5**); middle lane, co-spot of material on the left and right lanes; right lane, reaction mixture. The TLC shows that **5** was formed. Eluent: left TLC, hexanes/EtOAc 3:0.5; right TLC, EtOAc/MeOH 3:0.5.

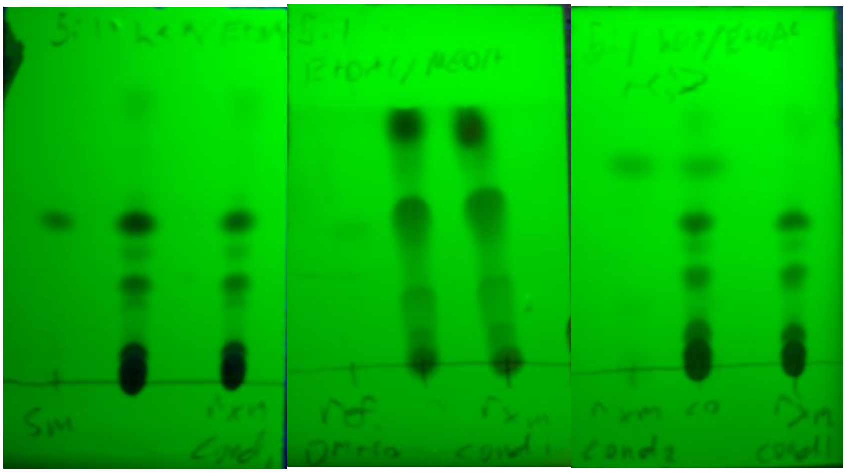

Figure S22. TLC for testing the stability of -$CH_2CH(SCH_2)_2CH_2$ group under Williamson ether formation conditions. Left TLC: left lane, $BnOCH_2CH$-$(SCH_2)_2CH_2$ (**3j**); middle lane, co-spot of materials on the left and right lanes; right lane, reaction mixture. The TLC shows that **3j** was not consumed. Middle TLC: left lane, DMTrO$(PEG)_8$OMe (**5**); middle lane, co-spot of materials on the left and right lanes; right lane, reaction mixture. The TLC shows that **5** was formed. Right TLC: left lane, reaction mixture of the β-elimination reaction of **3j**; middle lane, co-spot of materials on the left and right lanes; right lane, reaction mixture. The TLC shows that the β-elimination product of **3j** was not formed. Eluent: left TLC, hexanes/EtOAc 5:1; middle TLC, EtOAc/MeOH 5:1; right TLC, hexanes/EtOAc 5:1.

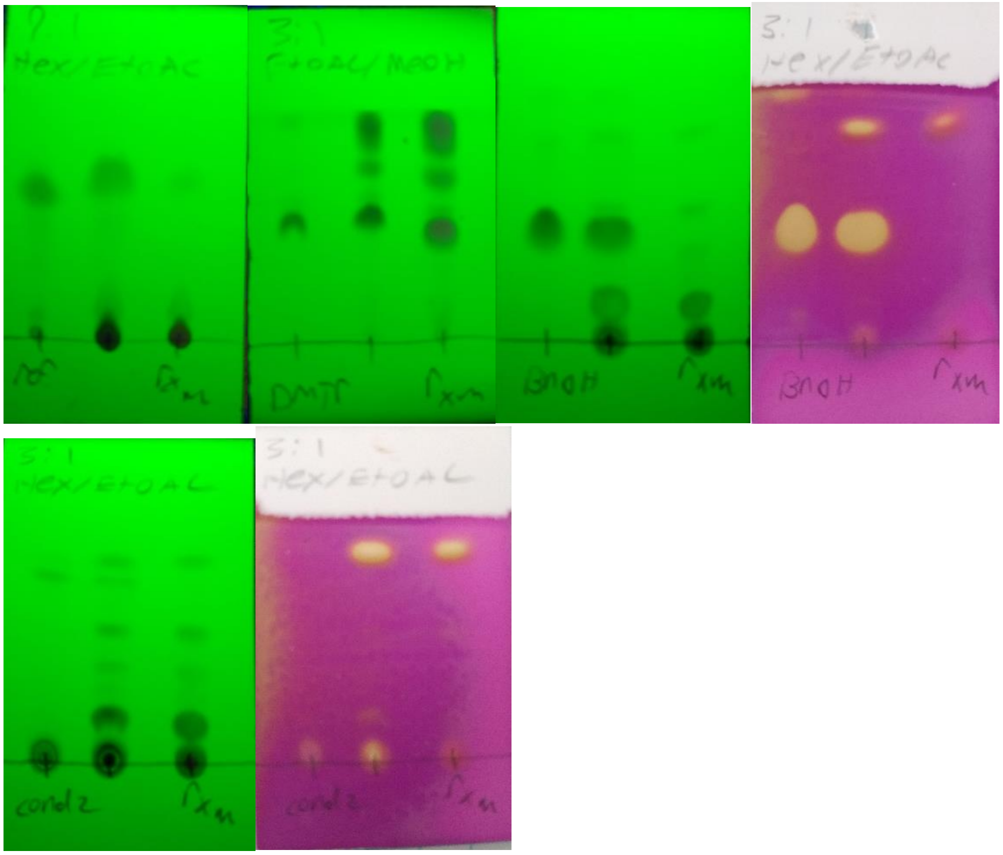

Figure S23. TLC for testing the stability of -$CH_2CH{=}CHMe$ group under Williamson ether formation conditions. Reading from left to right, first TLC: left lane, $BnOCH_2CH{=}CHMe$ (**3k**); middle lane, co-spot of materials on the left and right lanes; right lane, reaction mixture. The TLC shows that **3k** was not consumed. Second TLC: left lane, DMTrO(PEG)$_8$OMe (**5**); middle lane, co-spot of material on the left and right lanes; right lane, reaction mixture. The TLC shows that **5** was formed. Third TLC and fourth TLC ($KMnO_4$ stain): left lane, BnOH; middle lane, co-spot of materials on the left and right lanes; right lane, reaction mixture. The two TLCs show that β-elimination product was not formed. Fifth TLC and sixth TLC ($KMnO_4$ stain): left lane, reaction mixture of the β-elimination reaction of **3k**; middle lane, co-spot of materials on the left and right lanes; right lane, reaction mixture. The TLCs show that the β-elimination product of **3k** was not formed. Eluent: first TLC, hexanes/EtOAc 9:1; second TLC, EtOAc/MeOH 3:1; third and fourth TLCs, hexanes/EtOAc 3:1; fifth and sixth TLCs, hexanes/EtOAc 3:1.

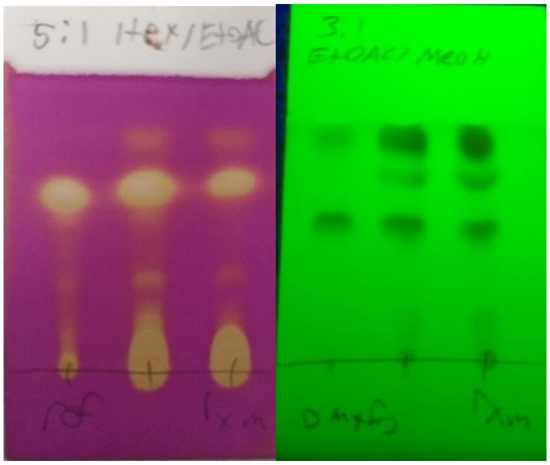

Figure S24. TLC for testing the stability of $-CH_2C{\equiv}CMe$ group under Williamson ether formation conditions. Left TLC: left lane, $EtOCH_2C{\equiv}CMe$ (**3l**); middle lane, co-spot of materials on the left and right lanes; right lane, reaction mixture. The TLC shows that the **3l** was not consumed. Right TLC: left lane, DMTrO(PEG)$_8$OMe (**5**); middle lane, co-spot of material on the left and right lanes; right lane, reaction mixture. The TLC shows that **5** was formed. Eluent: left TLC, hexanes/EtOAc 5:1; right TLC, EtOAc/MeOH 3:1.

TLC, NMR and MS of New Compounds

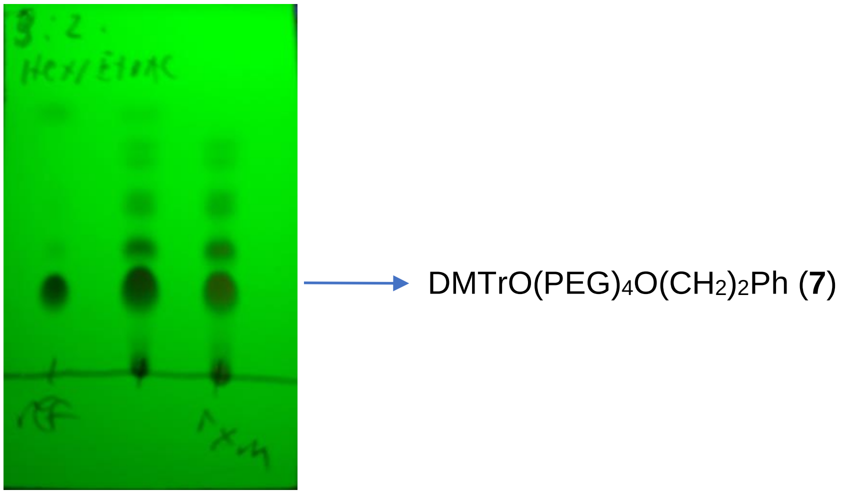

Figure S25. TLC of crude **7**. Eluent: hexanes/EtOAc 3:2. Left lane, purified **7**; middle lane, co-spot of materials on left and right lanes; right lane, crude **7**.

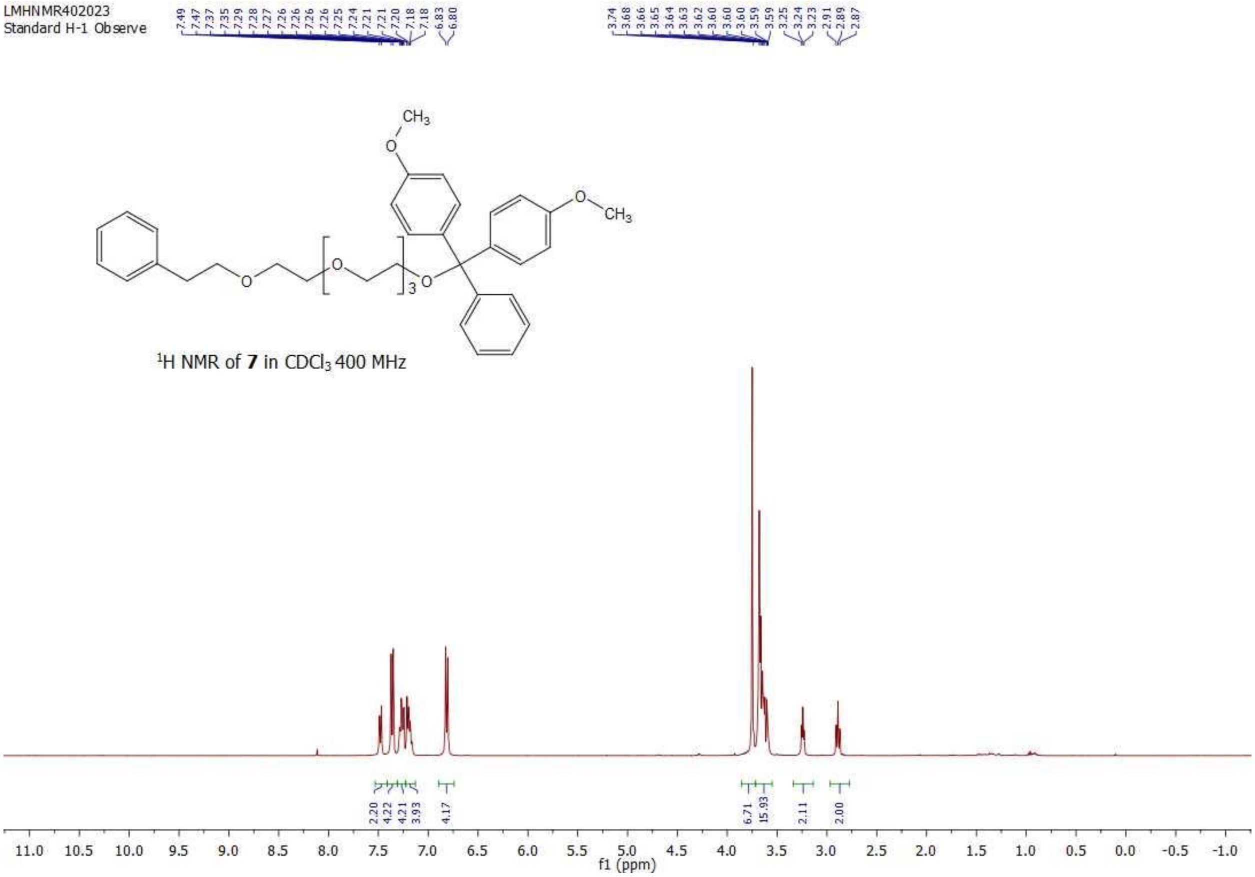

Figure S26. ^{1}H NMR of $Ph(CH_2)_2O(PEG)_4ODMTr$ (**7**).

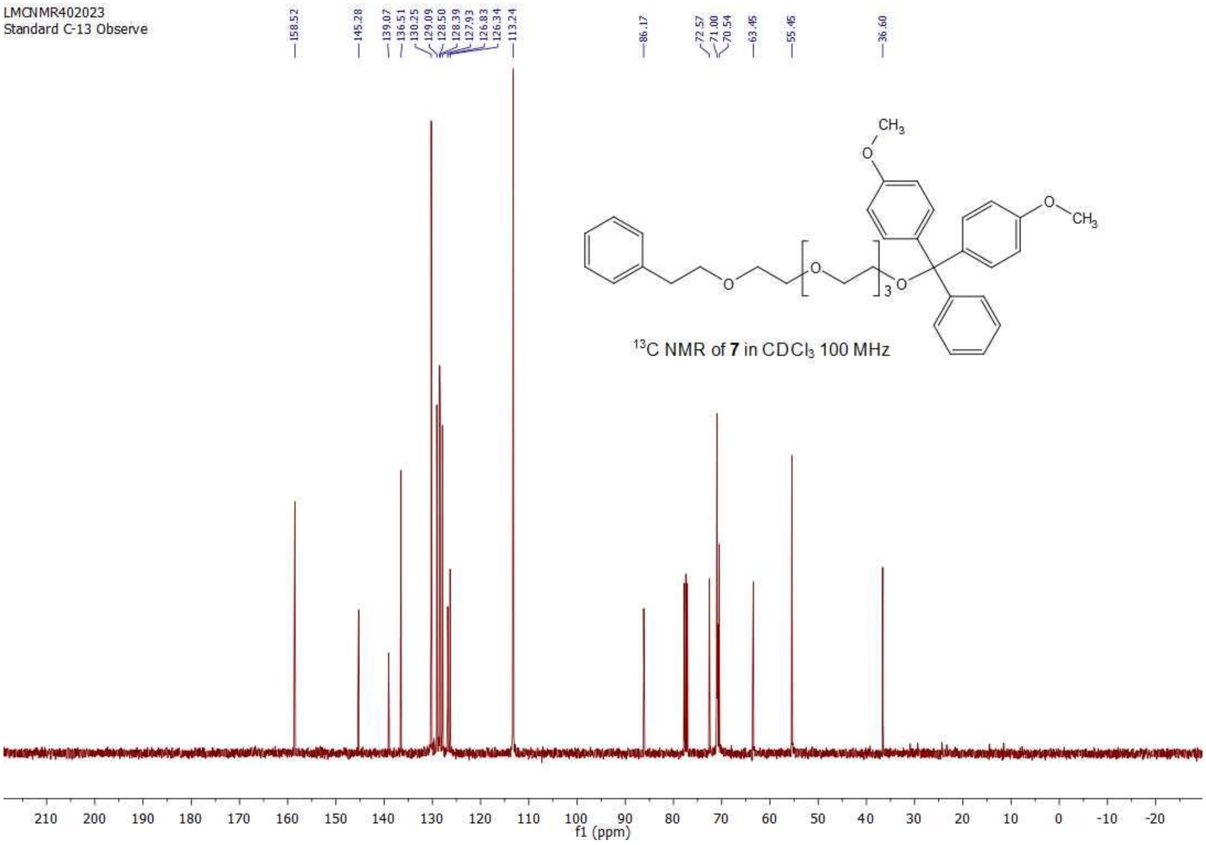

Figure S27. ^{13}C NMR of Ph(CH_2)$_2$O(PEG)$_4$ODMTr (**7**).

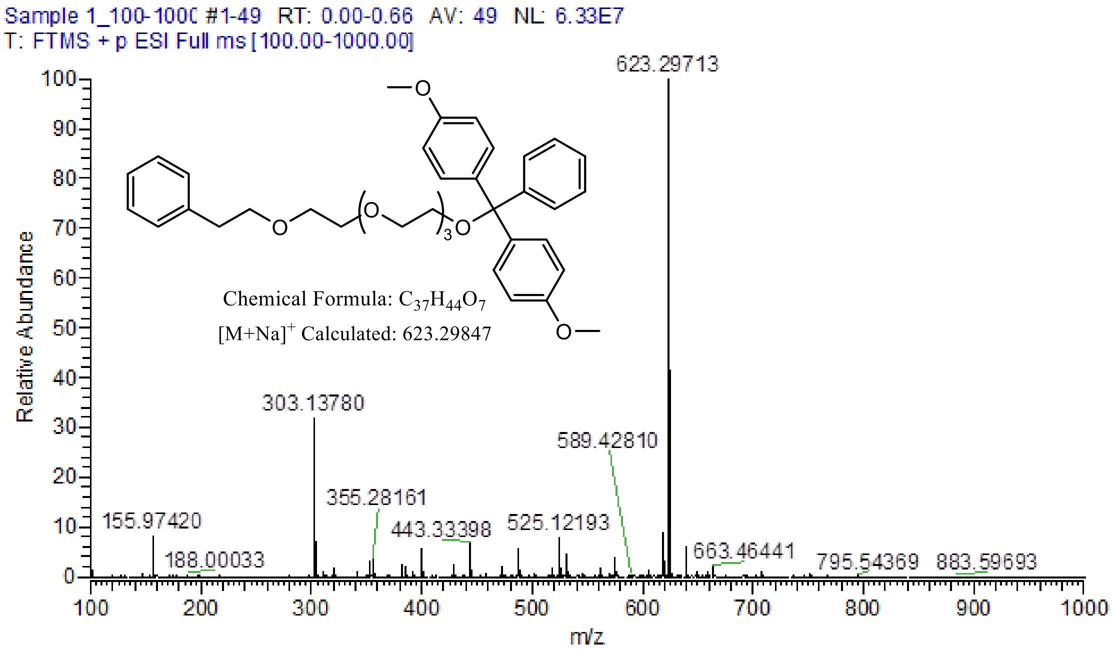

Figure S28. ESI-MS of Ph(CH_2)$_2$O(PEG)$_4$ODMTr (**7**).

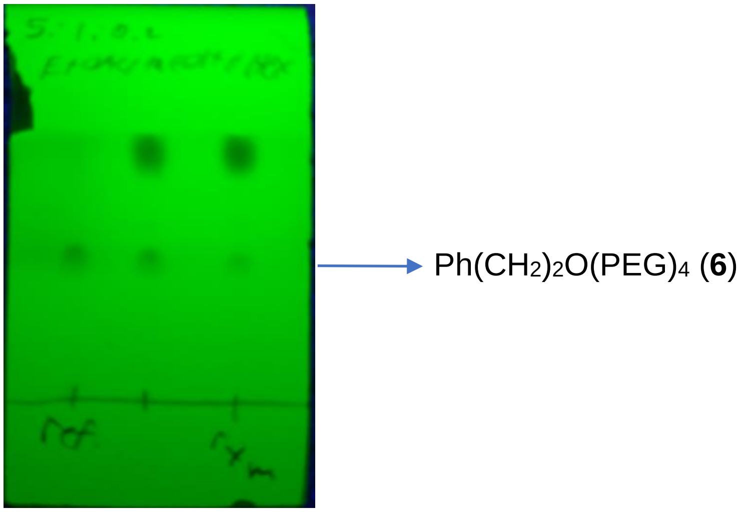

Figure S29. TLC of crude **6**. Eluent: EtOAc/MeOH/hexanes 5:1:0.2. Left lane, purified **6**; middle lane, co-spot of materials on left and right lanes; right lane, crude **6**.

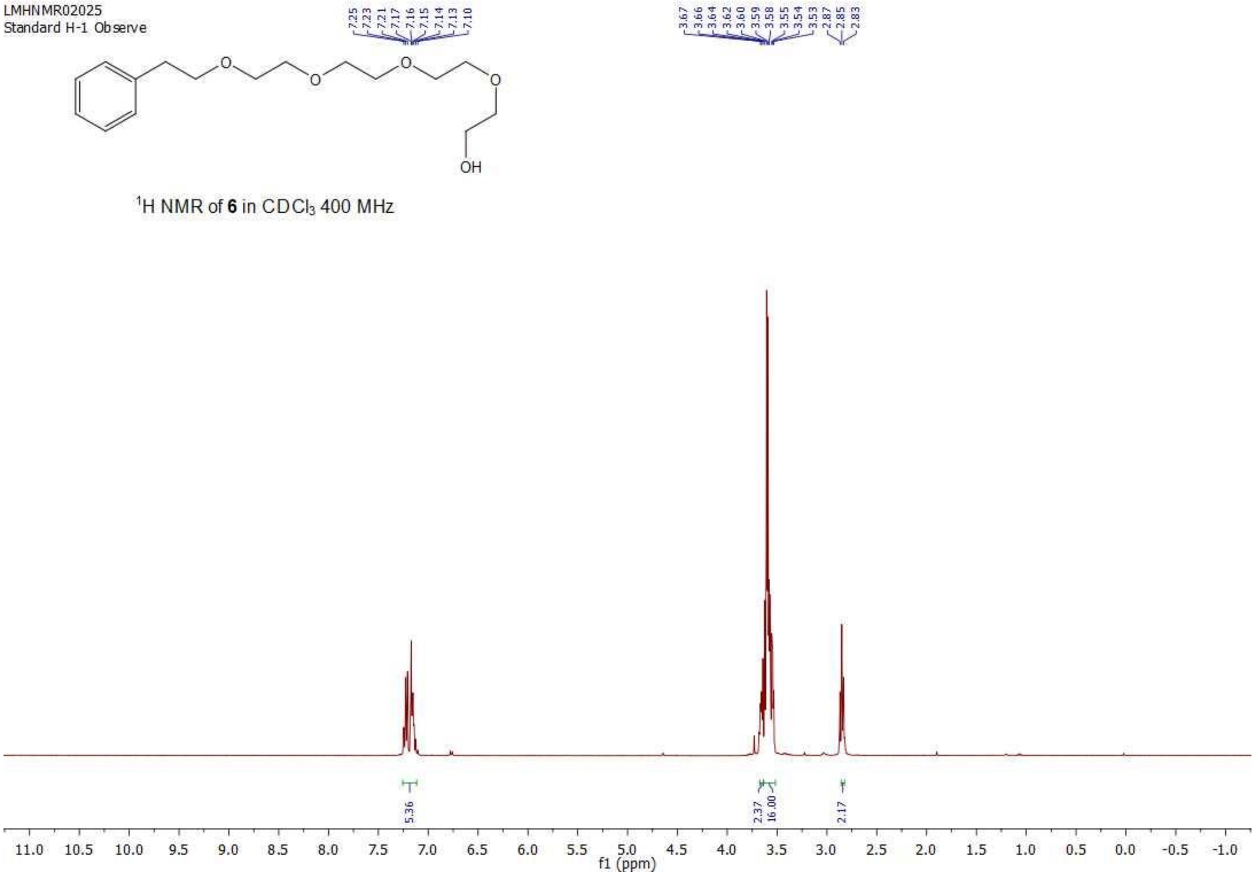

Figure S30. ^{1}H NMR of $Ph(CH_2)_2O(PEG)_4$ (**6**).

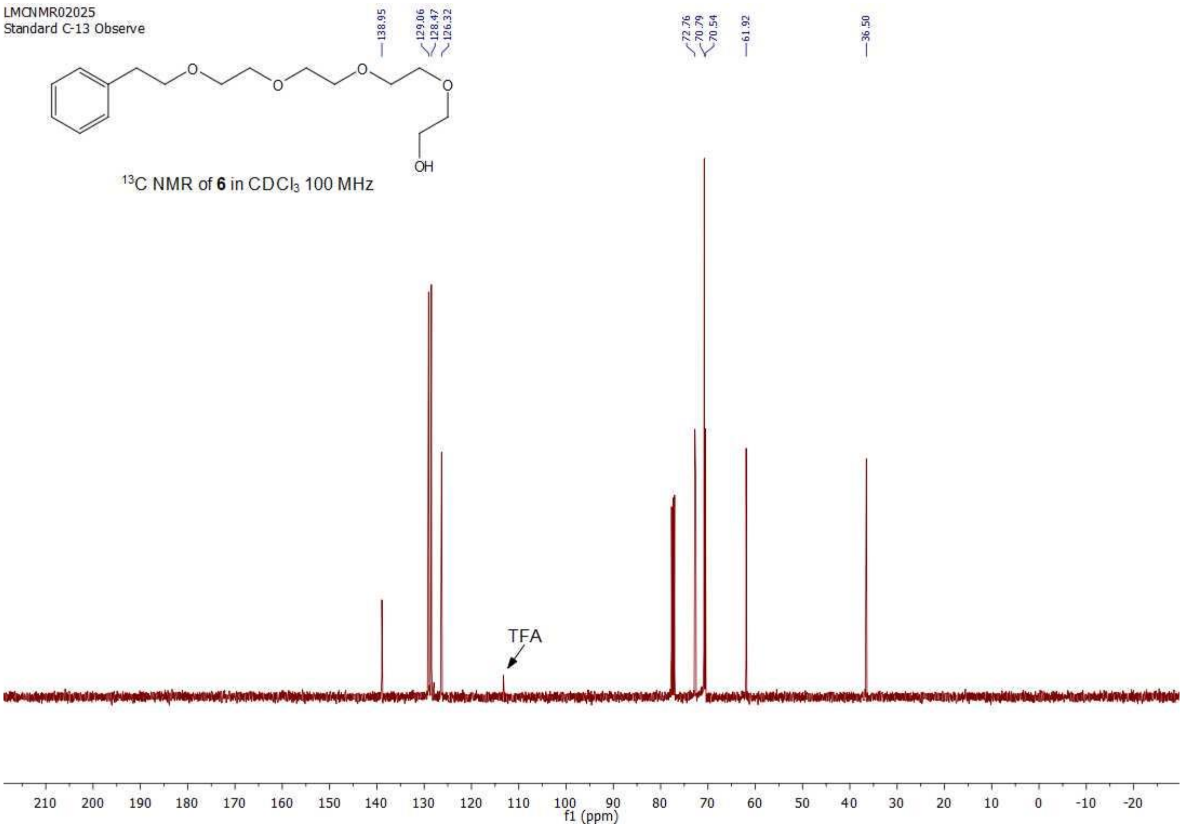

Figure S31. ^{13}C NMR of Ph(CH$_2$)$_2$O(PEG)$_4$ (**6**).

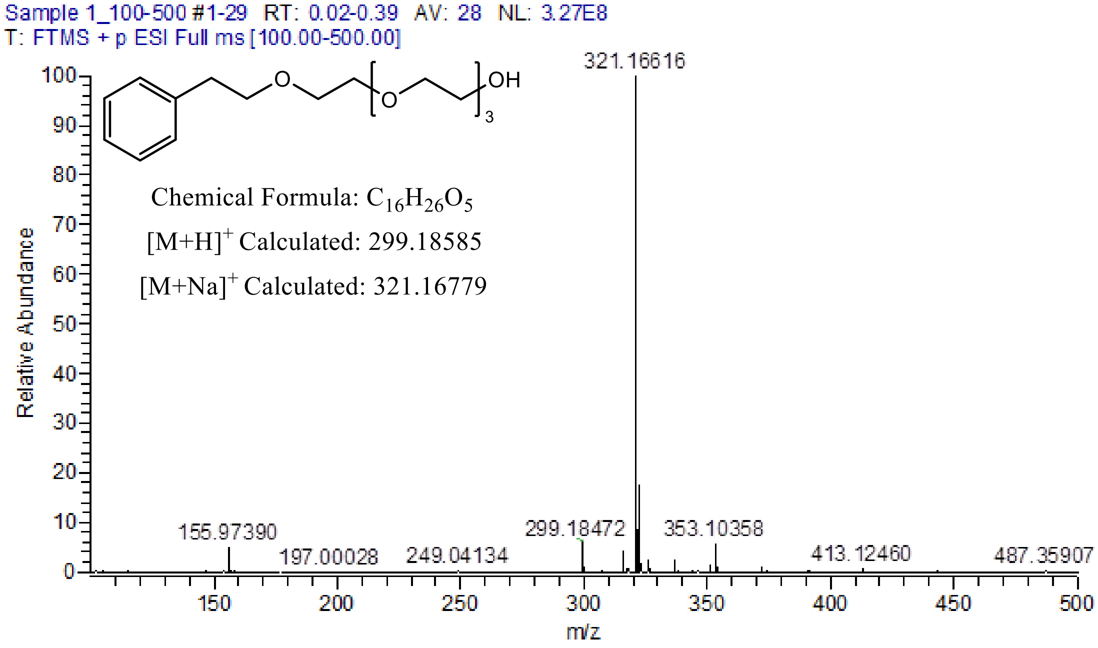

Figure S32. ESI-MS of Ph(CH$_2$)$_2$O(PEG)$_4$ (**6**).

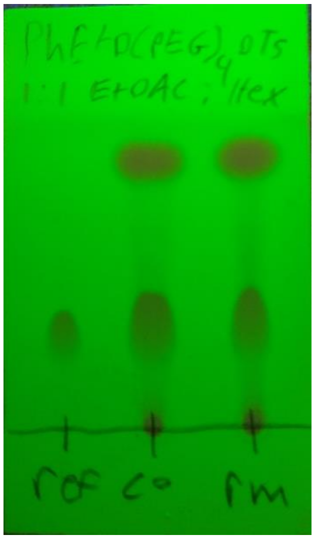

→ Ph(CH$_2$)$_2$O(PEG)$_4$OTs (**2**)

Figure S33. TLC of crude **2**. Eluent: hexanes/EtOAc 1:1. Left lane, purified **2**; middle lane, co-spot of materials on left and right lanes; right lane, crude **2**.

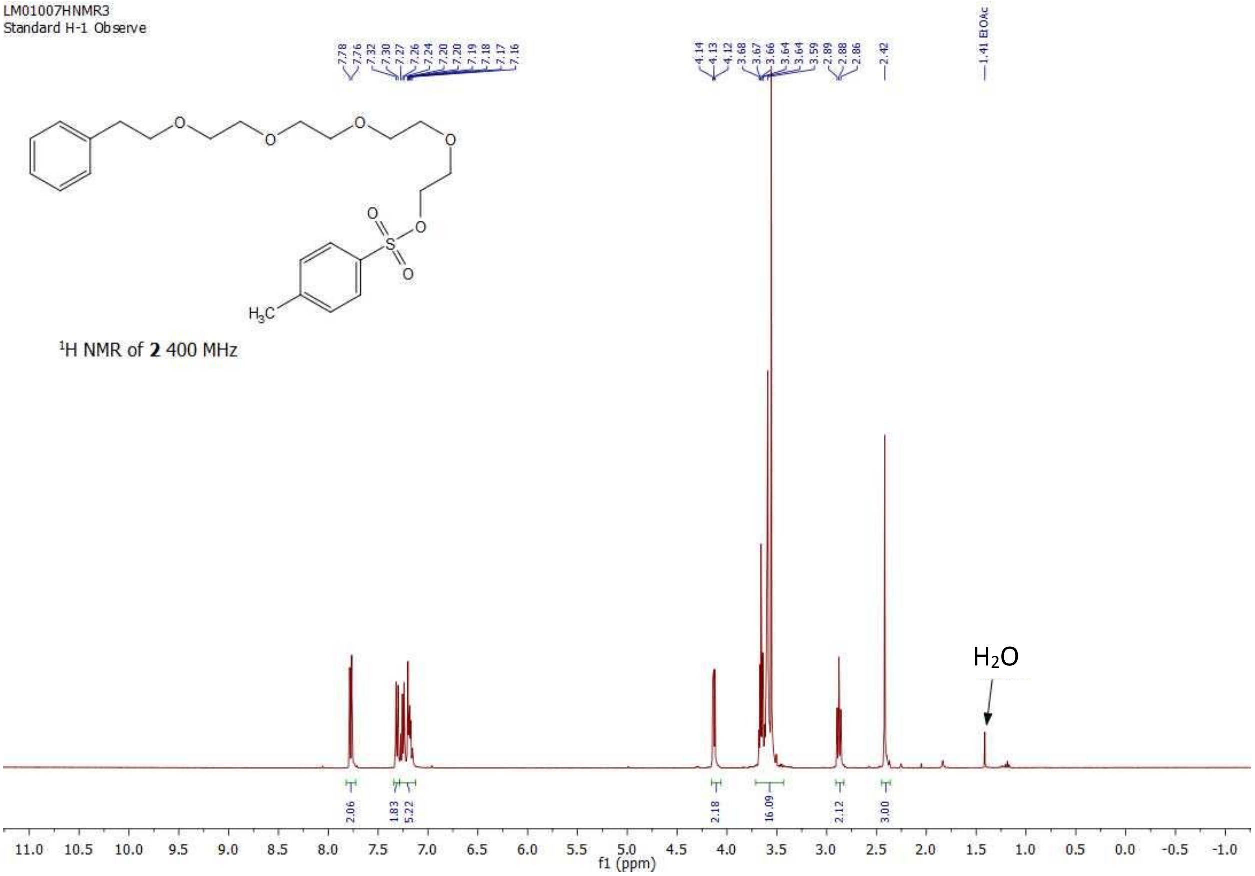

Figure S34. ^{1}H NMR of Ph(CH$_2$)$_2$O(PEG)$_4$OTs (**2**).

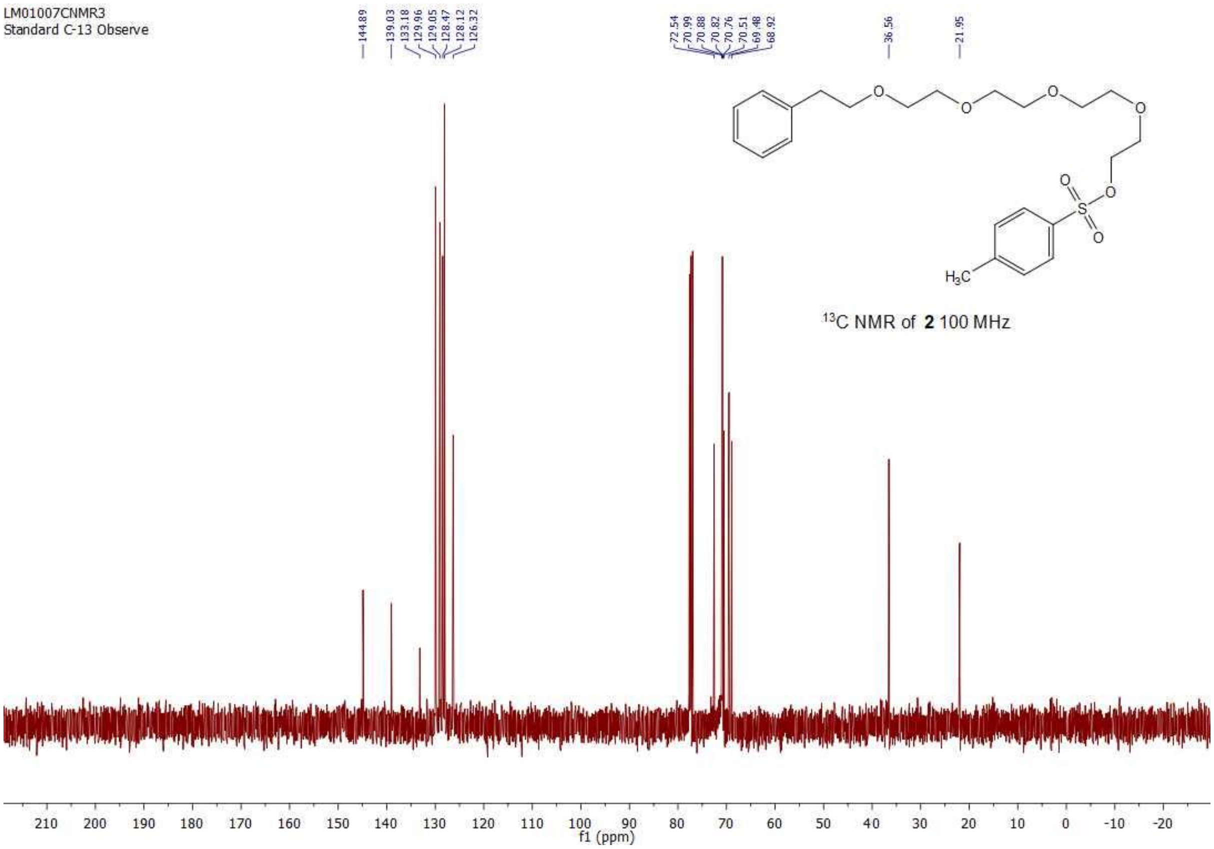

Figure S35. ^{13}C NMR of Ph$(CH_2)_2$O(PEG)$_4$OTs (**2**).

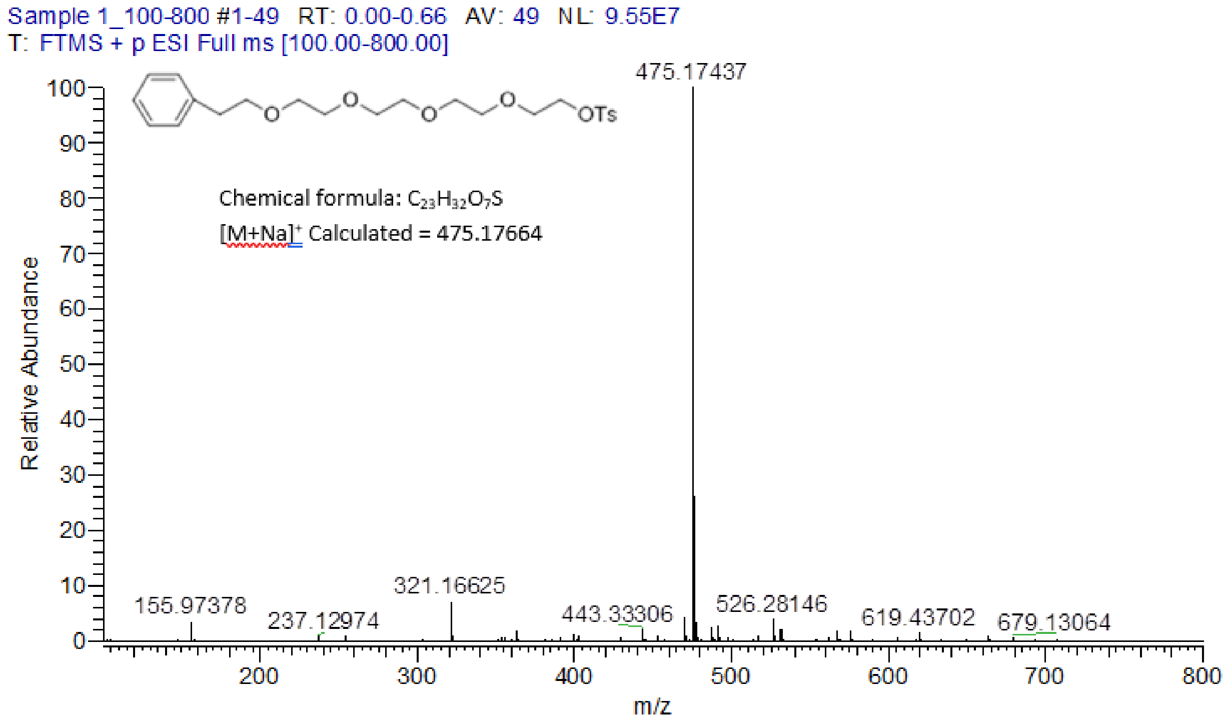

Figure S36. ESI-MS of Ph$(CH_2)_2$O(PEG)$_4$OTs (**2**).

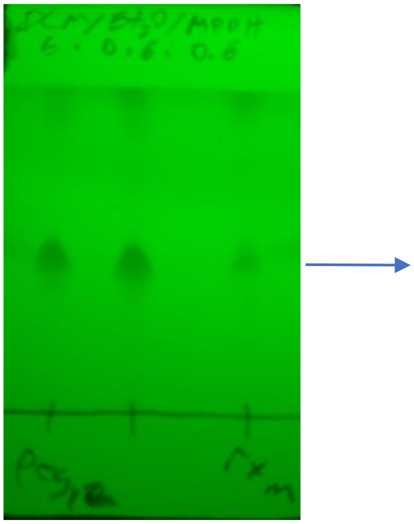

→ $Ph(CH_2)_2O(PEG)_{12}O(CH_2)_2Ph$ (**8**)

Figure S37. TLC of crude **8**. Eluent: DCM/MeOH/Et_2O 6:0.6:0.6. Left lane, purified **8**; co-spot of materials on left and right lanes; right lane, crude **8**.

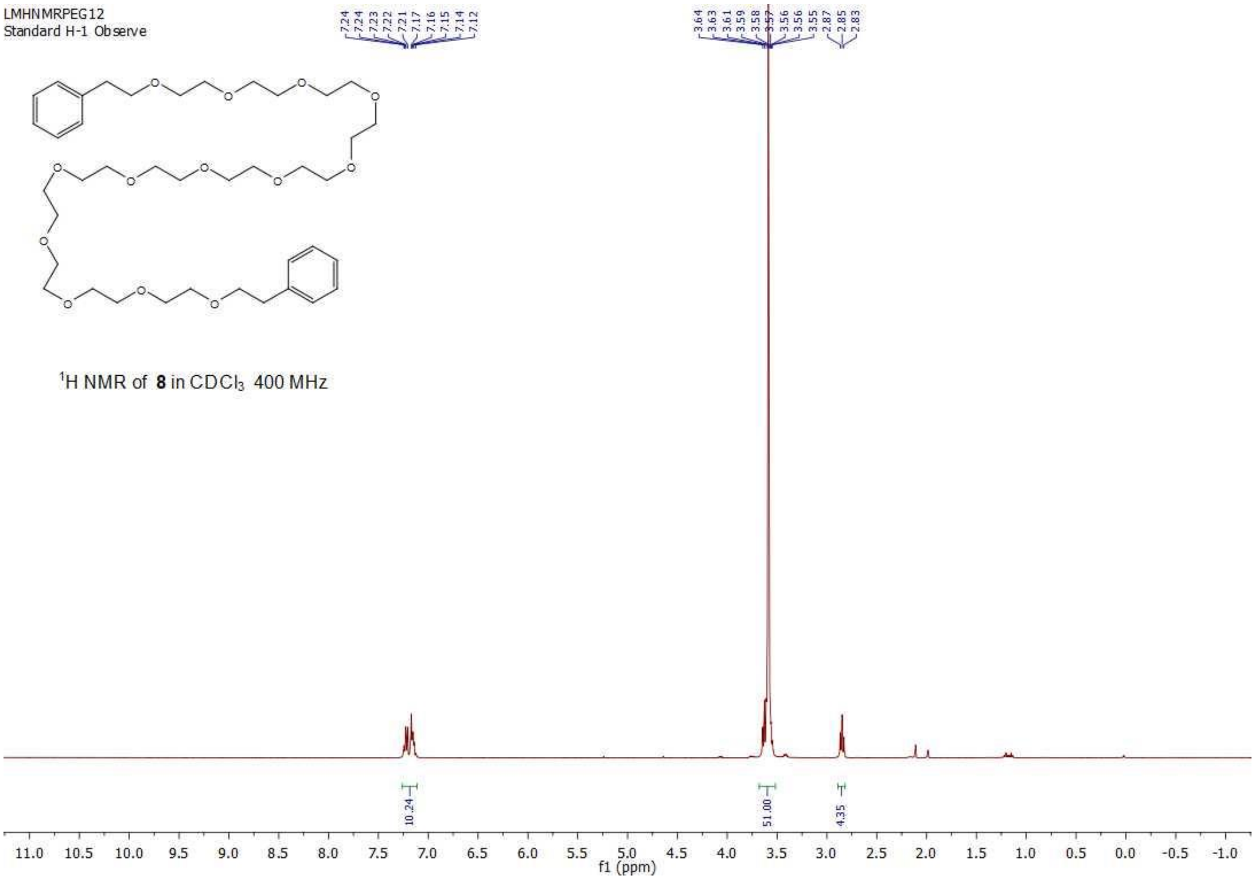

Figure S38. ^1H NMR of $Ph(CH_2)_2O(PEG)_{12}O(CH_2)_2Ph$ (**8**).

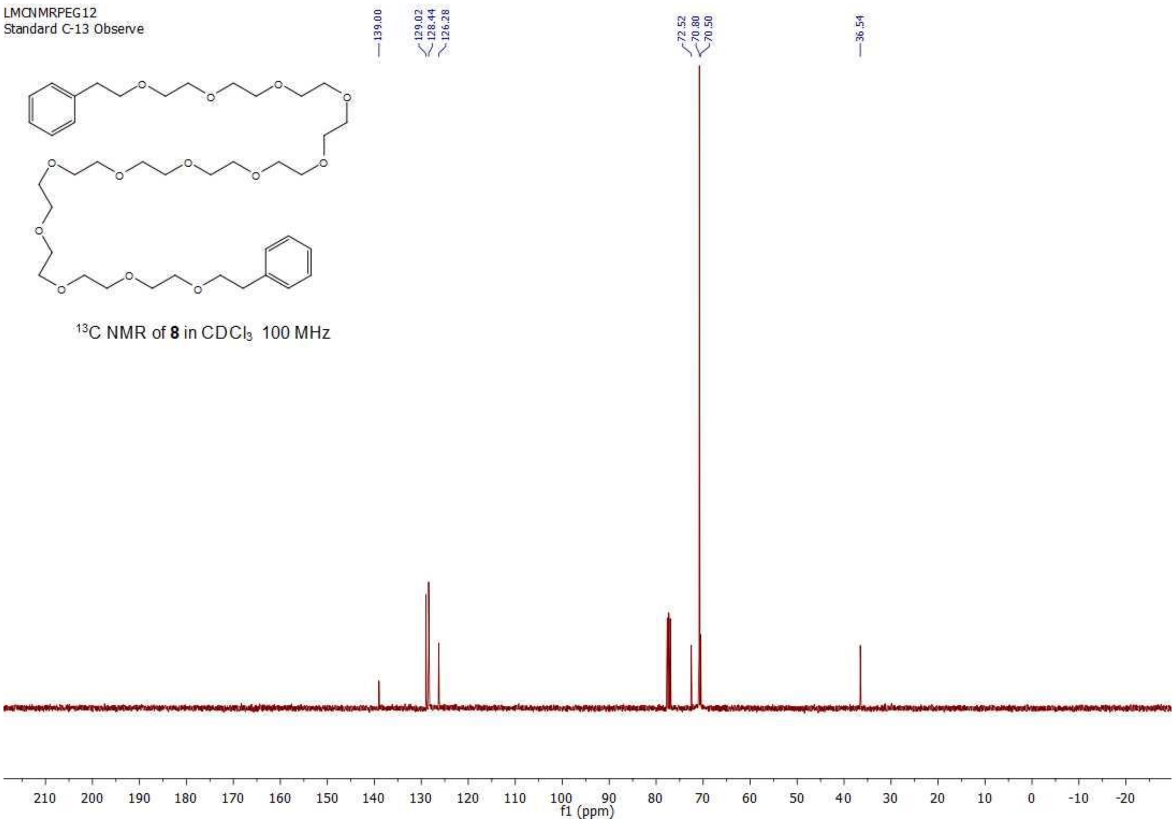

Figure S39. ^{13}C NMR of Ph(CH$_2$)$_2$O(PEG)$_{12}$O(CH$_2$)$_2$Ph (**8**).

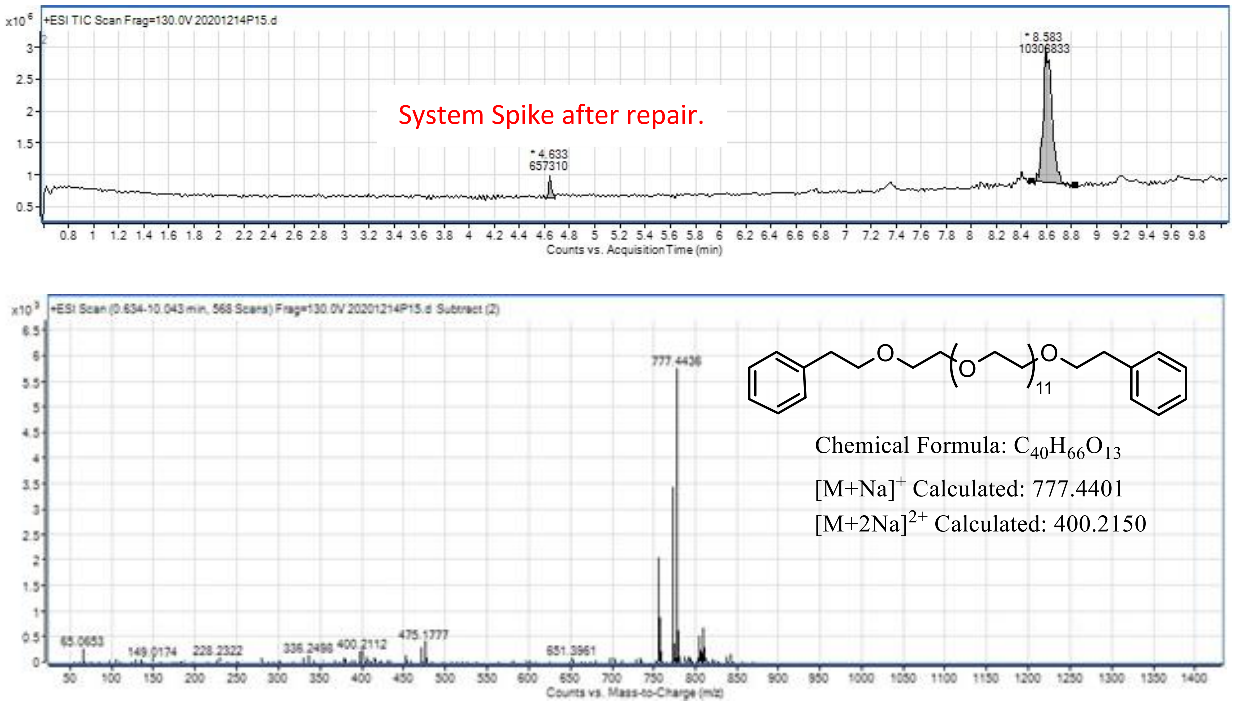

Figure S40. LC-MS of Ph(CH$_2$)$_2$O(PEG)$_{12}$O(CH$_2$)$_2$Ph (**8**).

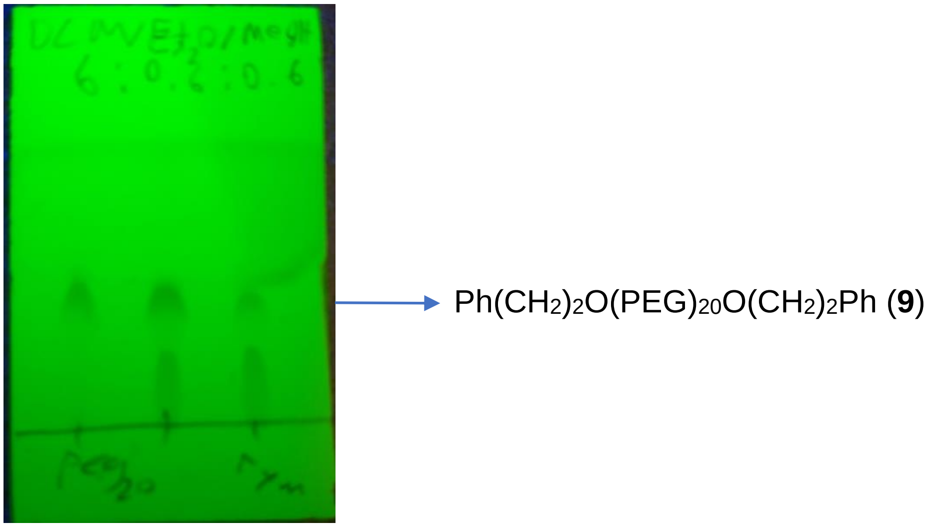

Figure S41. TLC of crude **9**. Eluent: DCM/MeOH/Et_2O 6:0.6:0.6. Left lane, purified **9**; co-spot of materials on left and right lanes; right lane, crude **9**.

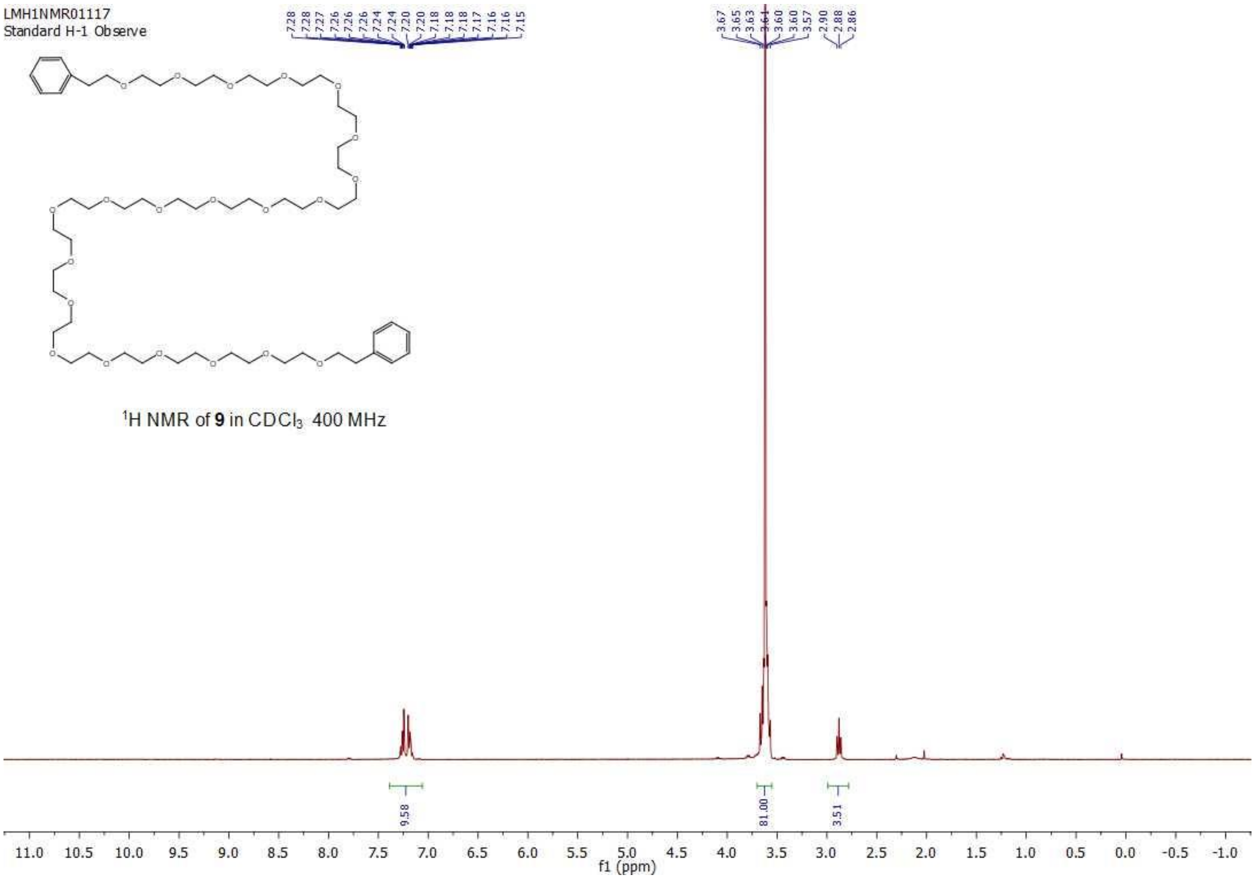

Figure S42. ^{1}H NMR of $Ph(CH_2)_2O(PEG)_{20}O(CH_2)_2Ph$ (**9**).

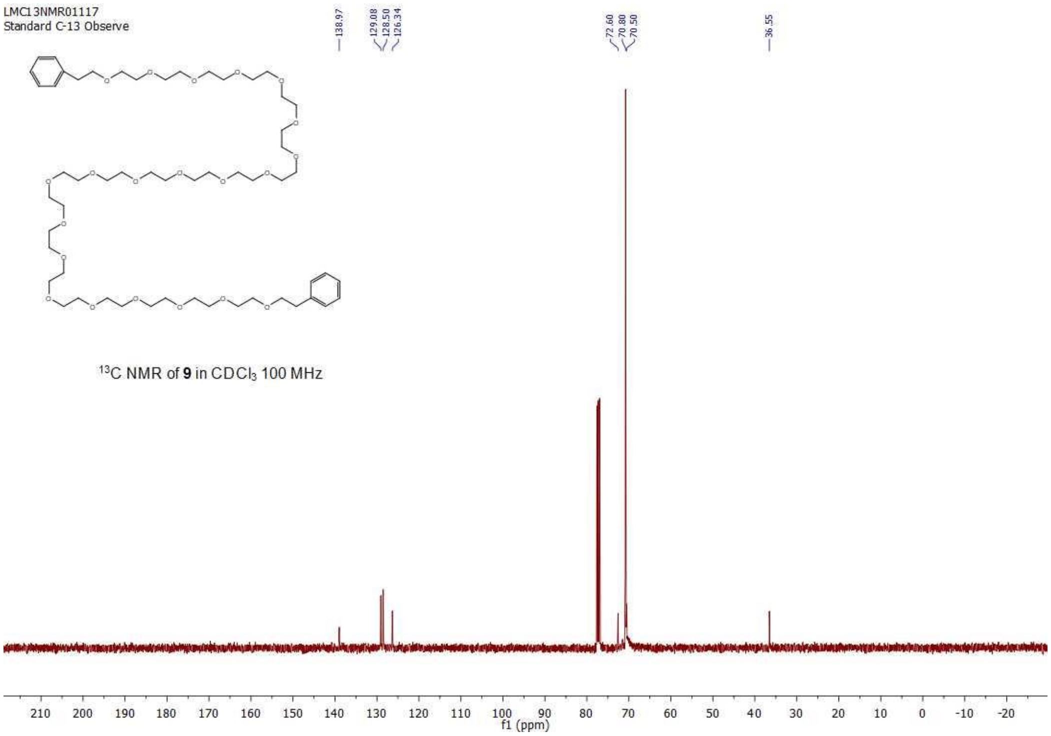

Figure S43. ^{13}C NMR of $Ph(CH_2)_2O(PEG)_{20}O(CH_2)_2Ph$ (**9**).

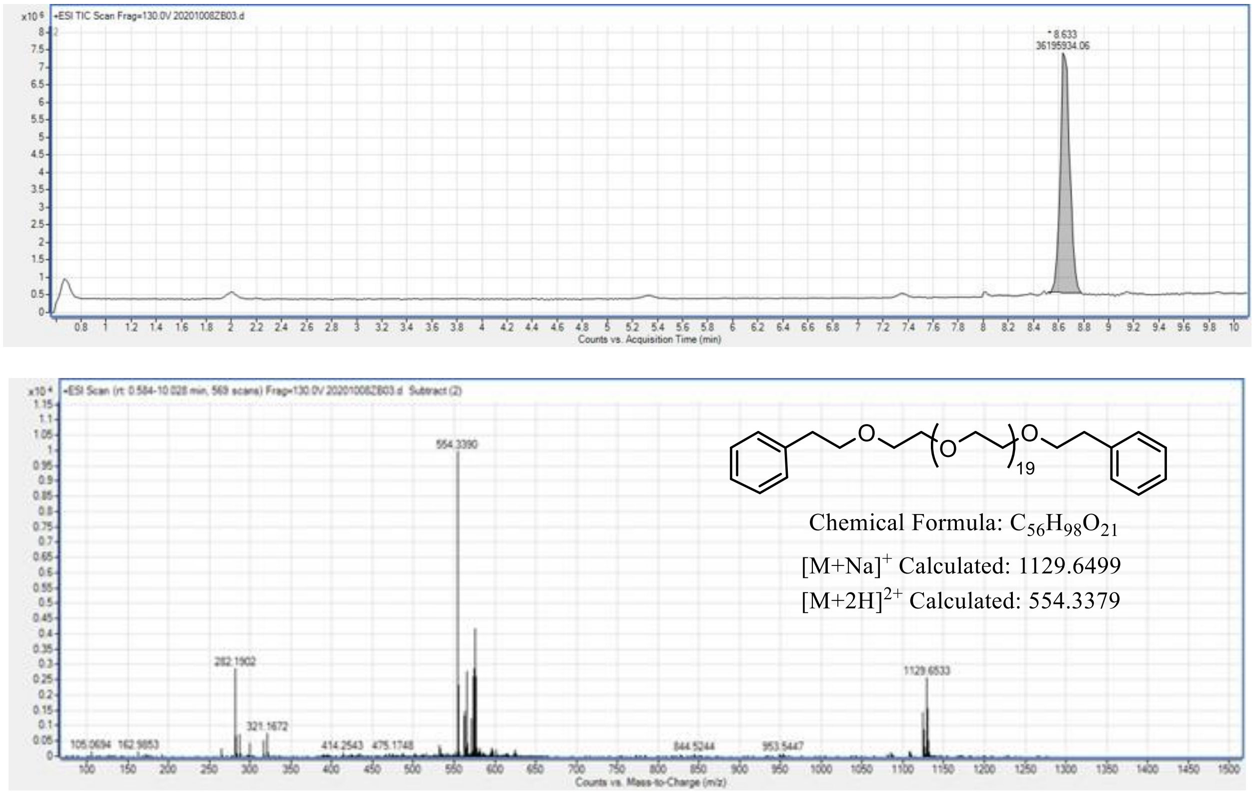

Figure S44. LC-MS of $Ph(CH_2)_2O(PEG)_{20}O(CH_2)_2Ph$ (**9**).

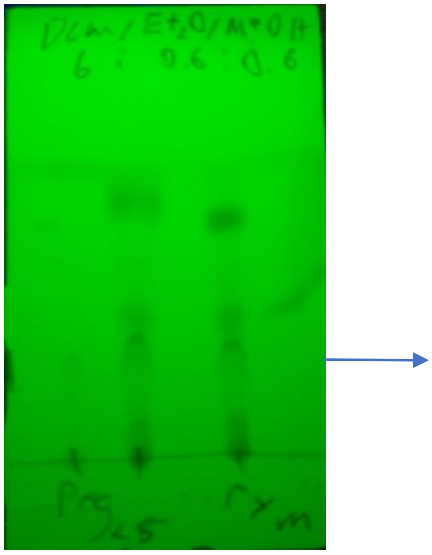

Ph(CH2)2O(PEG)28O(CH2)2Ph (**10**)

Figure S45. TLC of crude **10**. Eluent: DCM/MeOH/Et_2O 6:0.6:0.6. Left lane, purified **10**; middle lane, co-spot of materials on left and right lanes; right lane, crude **10**.

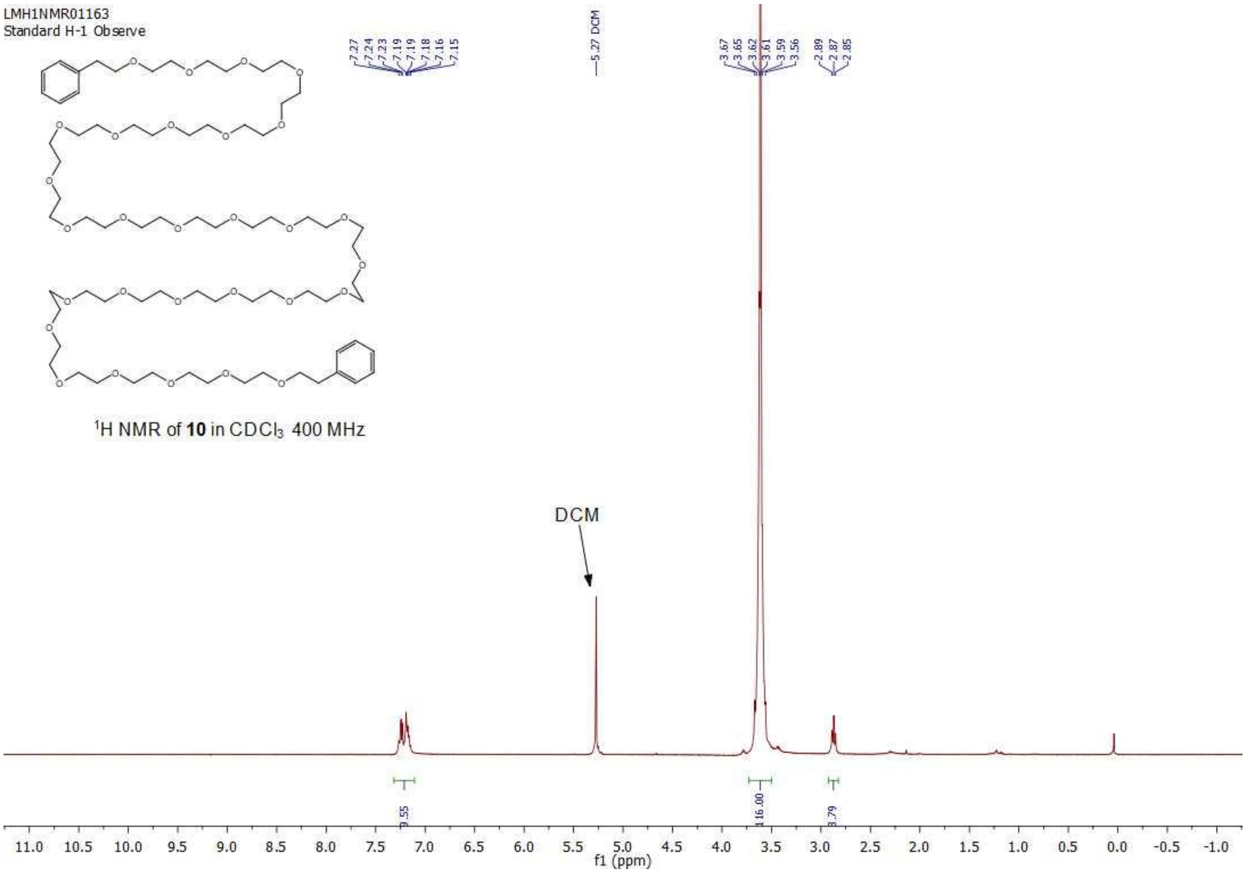

Figure S46. ^1H NMR of $Ph(CH_2)_2O(PEG)_{28}O(CH_2)_2Ph$ (**10**).

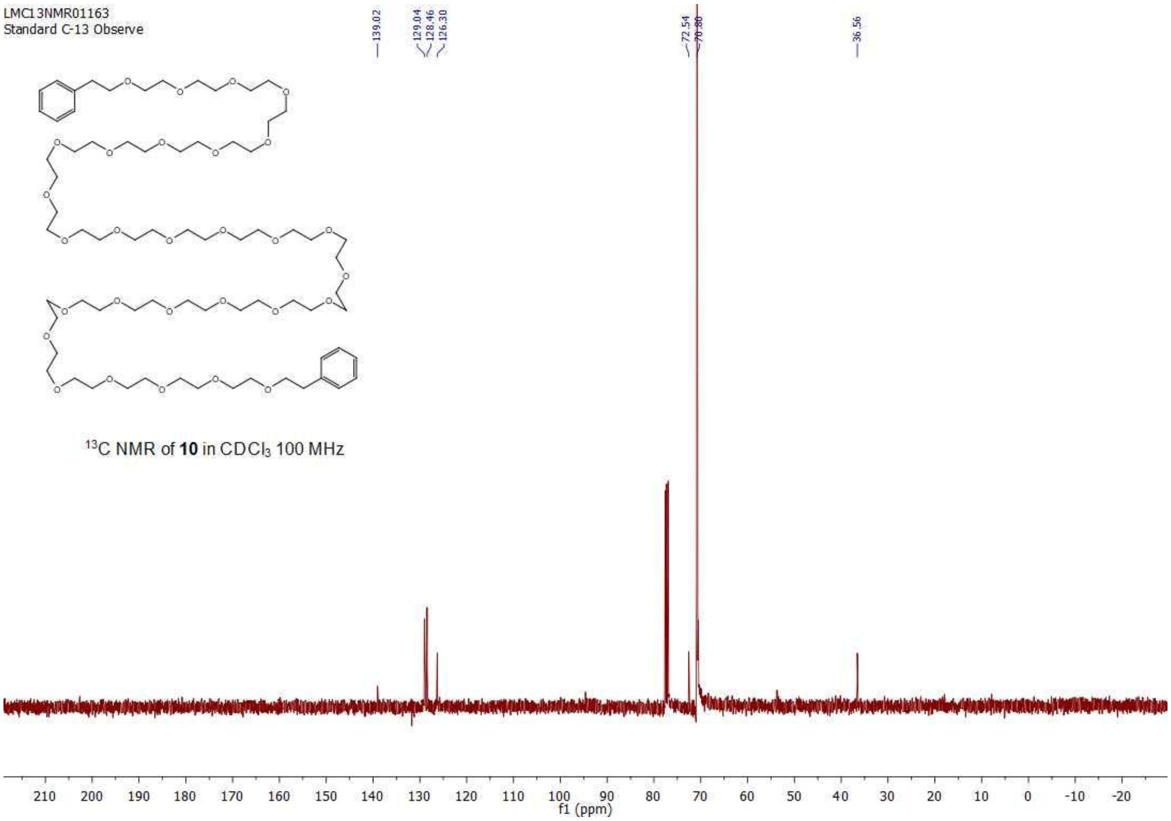

Figure S47. ^{13}C NMR of Ph(CH$_2$)$_2$O(PEG)$_{28}$O(CH$_2$)$_2$Ph (**10**).

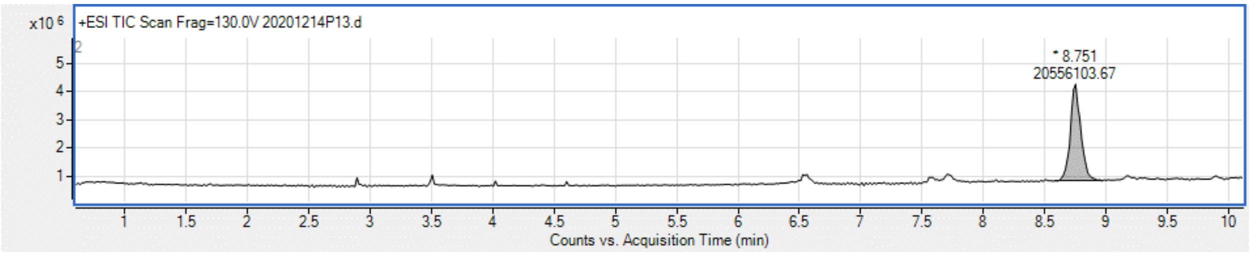

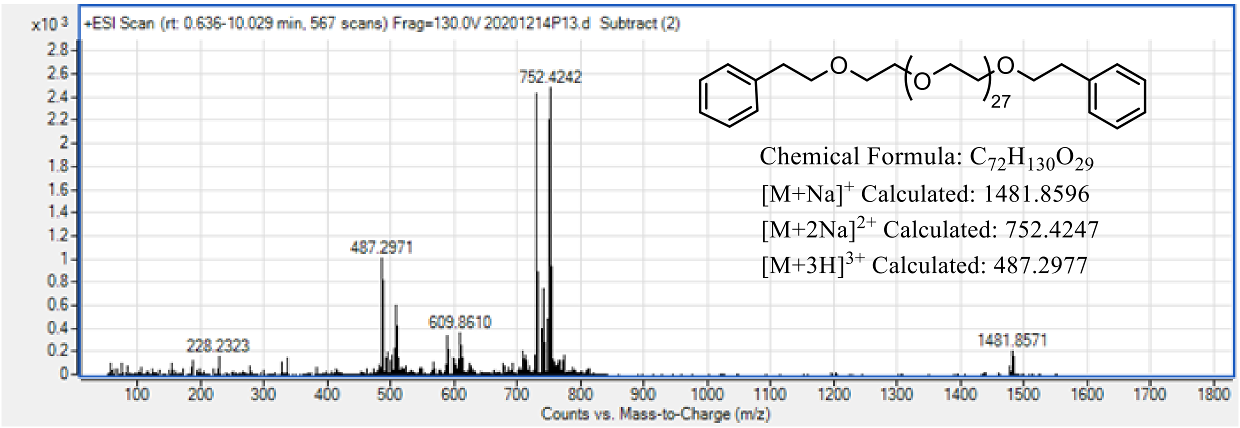

Figure S48. LC-MS of Ph(CH$_2$)$_2$O(PEG)$_{28}$O(CH$_2$)$_2$Ph (**10**).

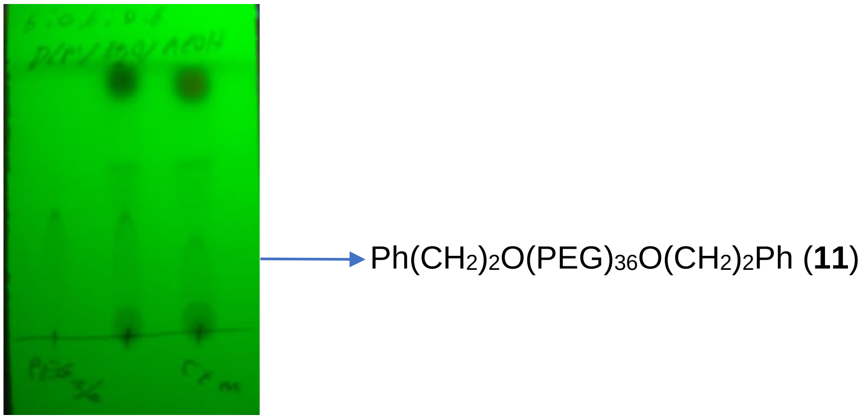

Figure S49. TLC of crude **11**. Eluent: DCM/MeOH/Et_2O 6:0.6:0.6. Left lane, starting material **10**; middle lane, co-spot of materials on the left and right lanes; right lane, crude **11**.

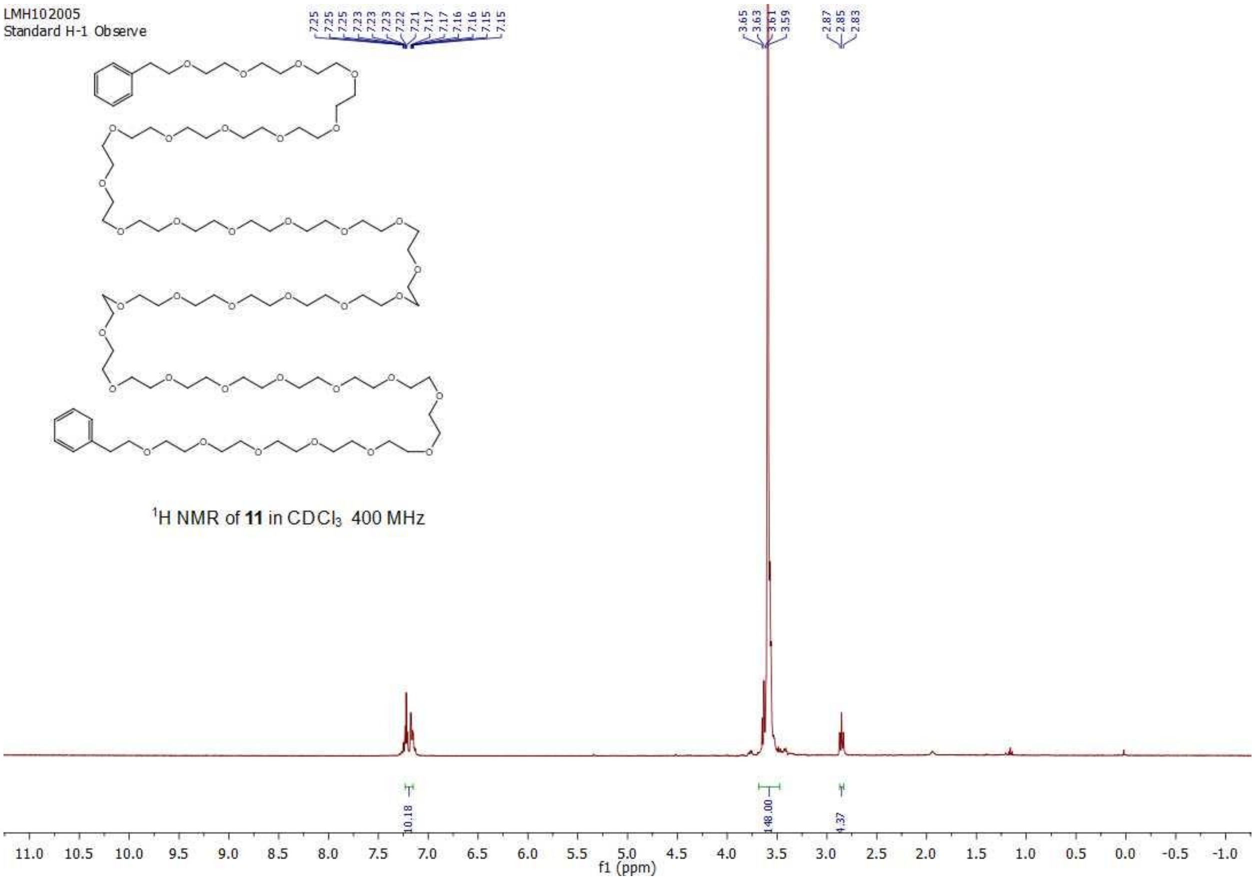

Figure S50. ^{1}H NMR of $Ph(CH_2)_2O(PEG)_{36}O(CH_2)_2Ph$ (**11**).

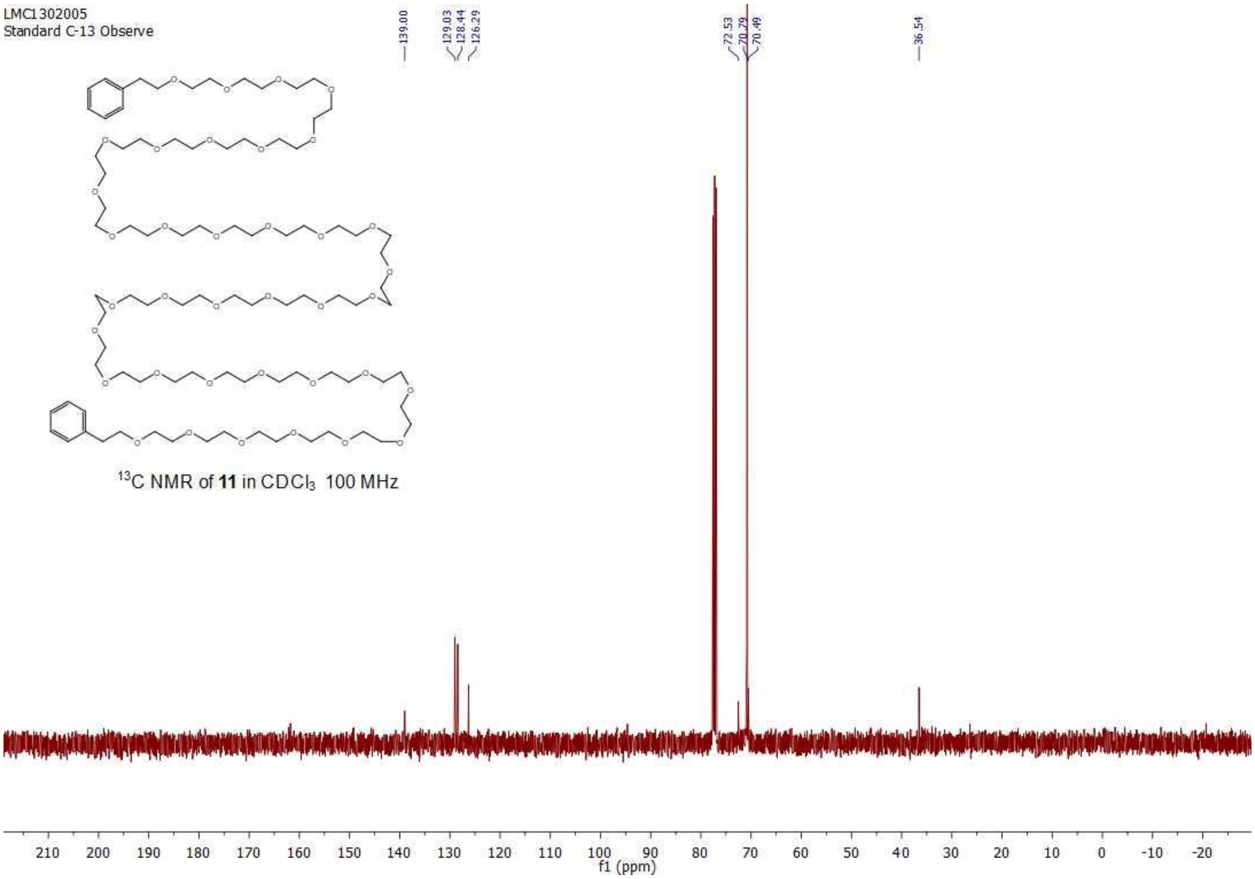

Figure S51. ^{13}C NMR of Ph(CH_2)$_2$O(PEG)$_{36}$O(CH_2)$_2$Ph (**11**).

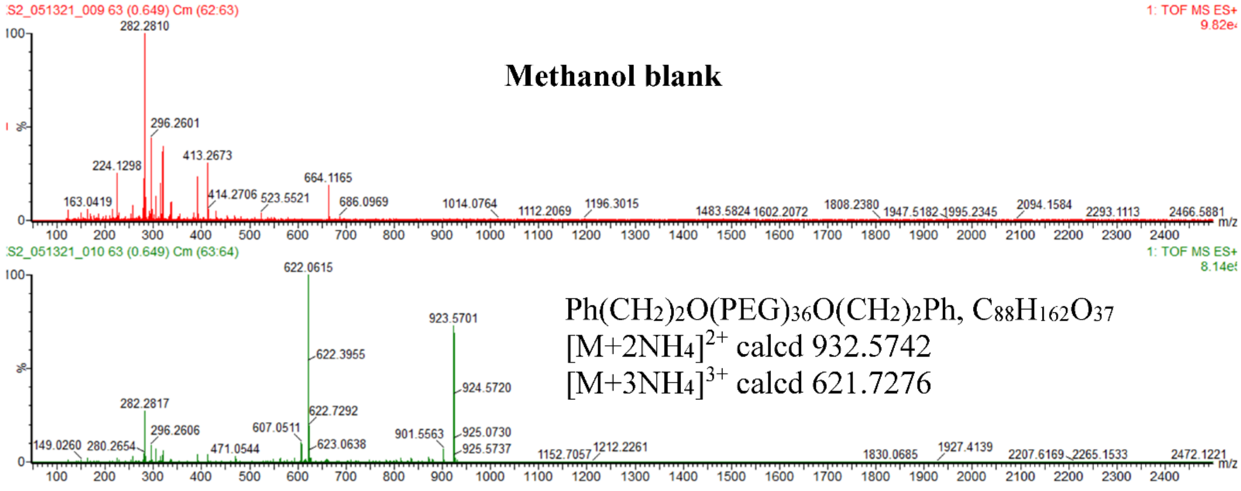

Figure S52. ESI-MS of Ph(CH_2)$_2$O(PEG)$_{36}$O(CH_2)$_2$Ph (**11**).

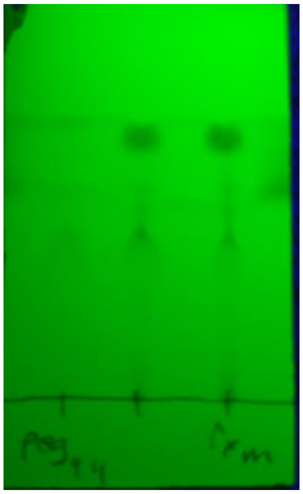

Ph(CH$_2$)$_2$O(PEG)$_{44}$O(CH$_2$)$_2$Ph (**12**)

Figure S53. TLC of crude **12**. Eluent: DCM/MeOH/Et$_2$O 6:1:1. Left lane, purified **12**; co-spot of materials on left and right lanes; right lane, crude **12**.

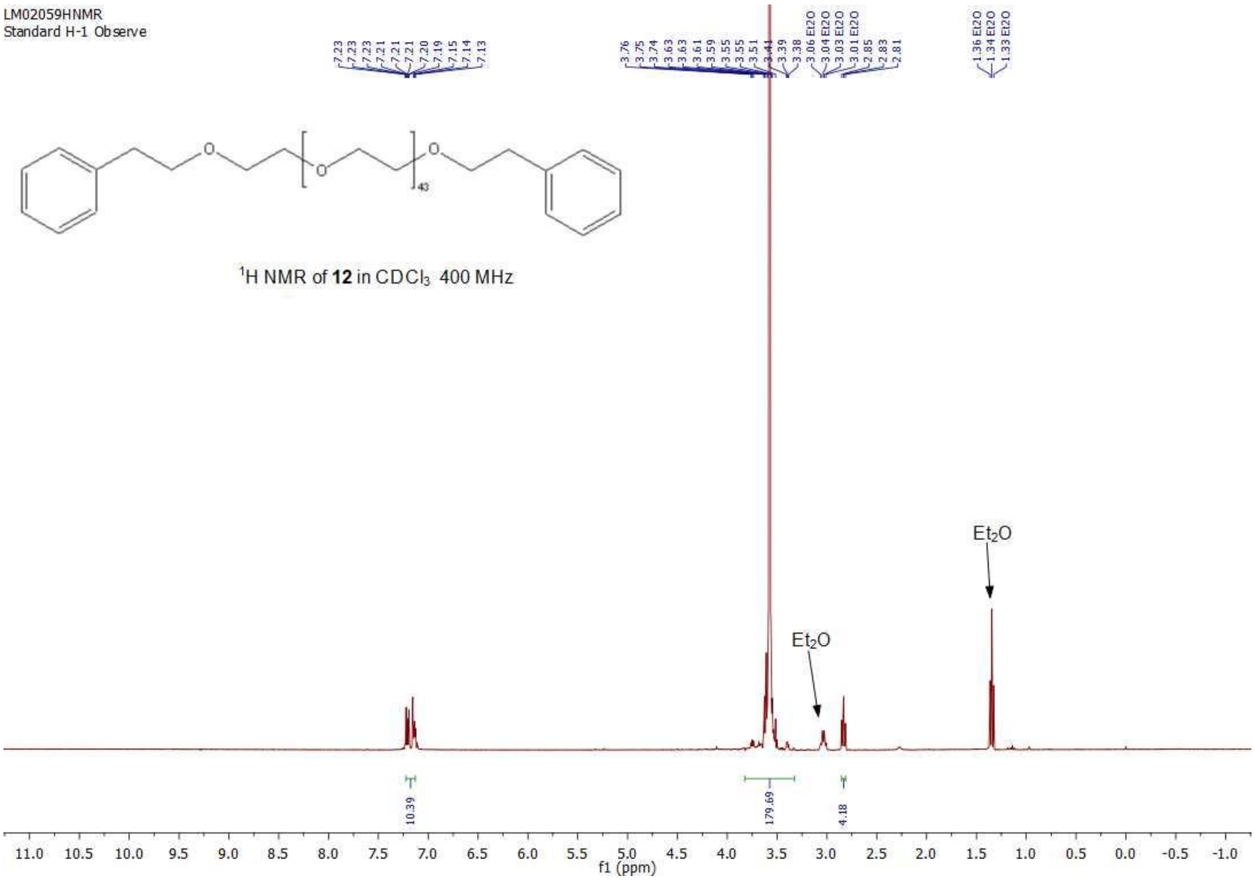

Figure S54. ^{1}H NMR of Ph(CH$_2$)$_2$O(PEG)$_{44}$O(CH$_2$)$_2$Ph (**12**).

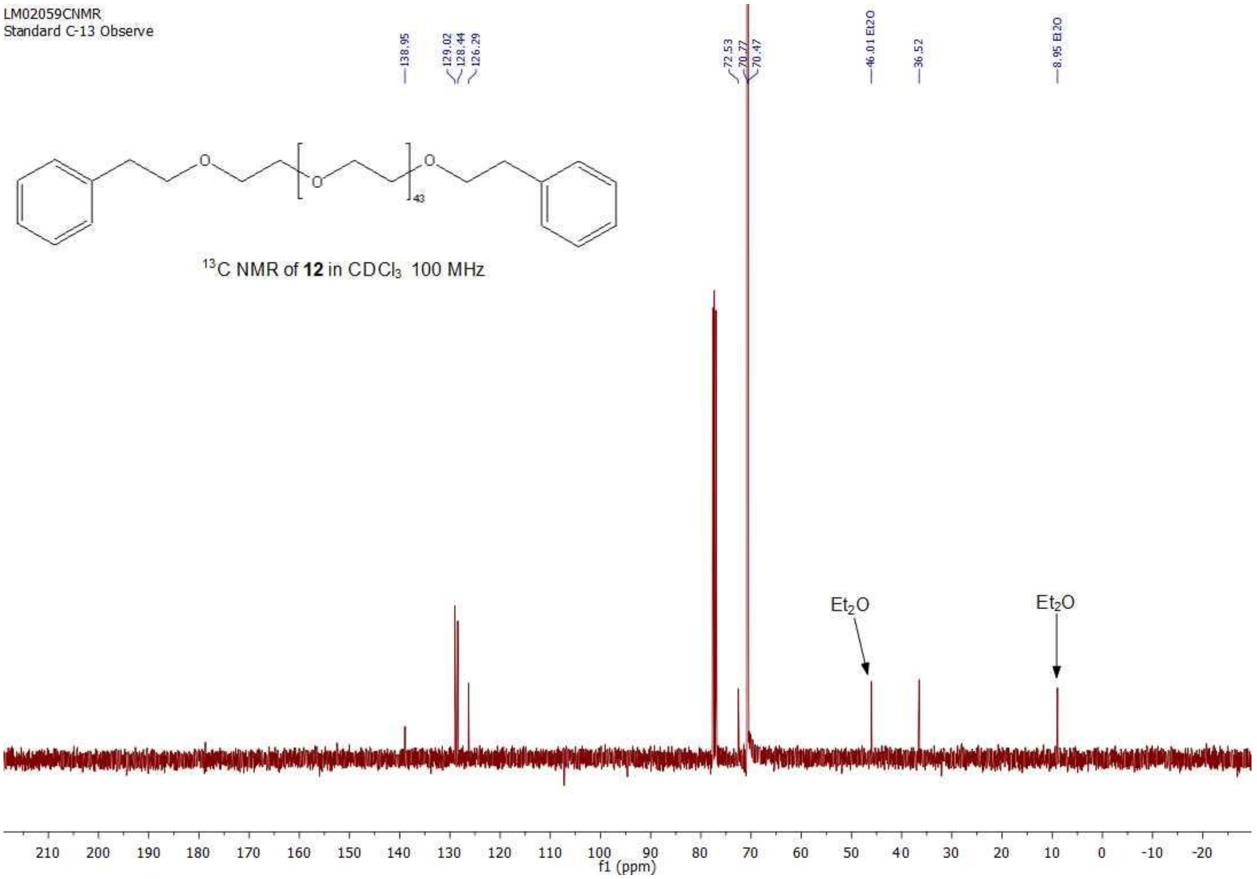

Figure S55. ^{13}C NMR of Ph(CH_2)$_2$O(PEG)$_{44}$O(CH_2)$_2$Ph (**12**).

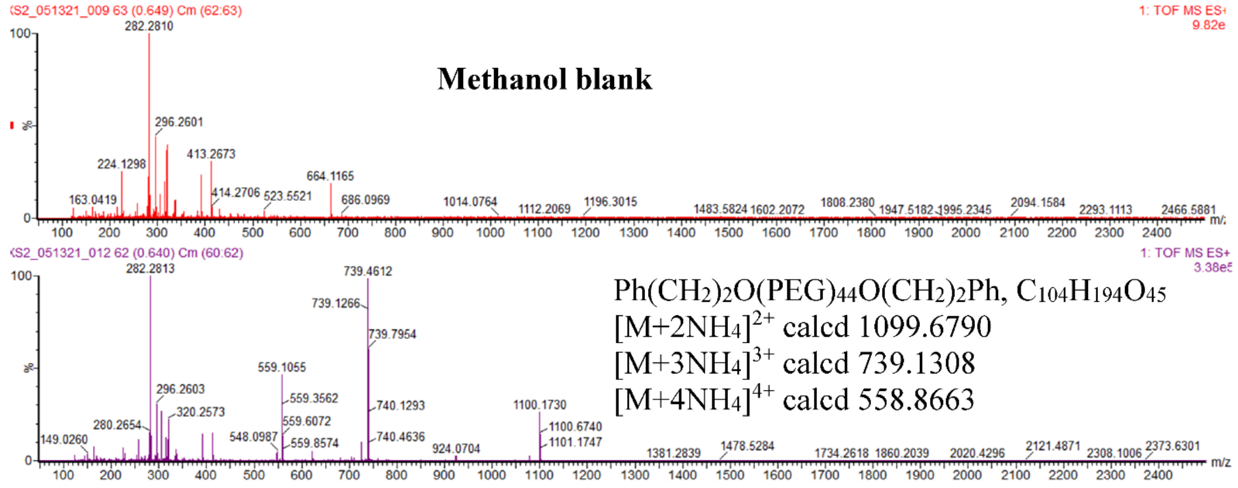

Figure S56. HRMS of Ph(CH_2)$_2$O(PEG)$_{44}$O(CH_2)$_2$Ph (**12**).

References

1. Rotstein, B. H.; Stephenson, N. A.; Vasdev, N.; Liang, S. H. *Nat. Commun.* **2014,** *5*. doi:10.1038/ncomms5365
2. Loiseau, F.; Simone, J. M.; Carcache, D.; Bobal, P.; Neier, R. *Monatshefte Fur Chemie* **2007,** *138*, 121-129. doi:10.1007/s00706-006-0578-x
3. Ilardi, E. A.; Stivala, C. E.; Zakarian, A. *Org. Lett.* **2008,** *10*, 1727-1730. doi:10.1021/ol800341z
4. Margot, C.; Rizzolio, M.; Schlosser, M. *Tetrahedron* **1990,** *46*, 2411-2424. doi:10.1016/S0040-4020(01)82022-6
5. Margot, C.; Matsuda, H.; Schlosser, M. *Tetrahedron* **1990,** *46*, 2425-2430. doi:10.1016/S0040-4020(01)82023-8
6. Khanal, A.; Fang, S. *Chem. Eur. J.* **2017,** *23*, 15133-15142. doi:10.1002/chem.201703004
7. Ouchi, M.; Inoue, Y.; Liu, Y.; Nagamune, S.; Nakamura, S.; Wada, K.; Hakushi, T. *Bull. Chem. Soc. Jpn.* **1990,** *63*, 1260-1262. doi:10.1246/bcsj.63.1260
